# Blood metabolites and chronic kidney disease: a Mendelian randomization study

**DOI:** 10.1186/s12920-024-01918-3

**Published:** 2024-05-28

**Authors:** Yawei Hou, Zhenwei Xiao, Yushuo Zhu, Yameng Li, Qinglin Liu, Zhenguo Wang

**Affiliations:** 1 Institute of Chinese Medical Literature and Culture, Shandong University of Traditional Chinese Medicine, Jinan, China; 2 Department of Nephrology, Affiliated Hospital of Shandong University of Traditional Chinese Medicine, Jinan, China; 3 Department of Emergency and Critical Care Medicine, Affiliated Hospital of Shandong University of Traditional Chinese Medicine, Jinan, China; 4 The First Clinical Medical College, Shandong University of Traditional Chinese Medicine, Jinan, China; 5 College of Traditional Chinese Medicine, Shandong University of Traditional Chinese Medicine, Jinan, China

**Keywords:** Mendelian randomization, Chronic kidney disease, Renal function, Human blood metabolites

## Abstract

**Background:**

Human blood metabolites have demonstrated close associations with chronic kidney disease (CKD) in observational studies. Nonetheless, the causal relationship between metabolites and CKD is still unclear. This study aimed to assess the associations between metabolites and CKD risk.

**Methods:**

We applied a two-sample Mendelian randomization (MR) analysis to evaluate relationships between 1400 blood metabolites and eight phenotypes (outcomes) (CKD, estimated glomerular filtration rate(eGFR), urine albumin to creatinine ratio, rapid progress to CKD, rapid decline of eGFR, membranous nephropathy, immunoglobulin A nephropathy, and diabetic nephropathy). The inverse variance weighted (IVW), MR-Egger, and weighted median were used to investigate the causal relationship. Sensitivity analyses were performed with Cochran’s Q, MR-Egger intercept, MR-PRESSO Global test, and leave-one-out analysis. Bonferroni correction was used to test the strength of the causal relationship.

**Results:**

Through the MR analysis of 1400 metabolites and eight clinical phenotypes, a total of 48 metabolites were found to be associated with various outcomes. Among them, N-acetylleucine (OR = 0.923, 95%CI: 0.89–0.957, *P*_IVW_ = 1.450 × 10^–5^) has a strong causal relationship with lower risk of CKD after the Bonferroni-corrected test, whereas Glycine to alanine ratio has a strong causal relationship with higher risk of CKD (OR = 1.106, 95%CI: 1.063–1.151,* P*_IVW_ = 5.850 × 10^–7^). No horizontal pleiotropy and heterogeneity were detected.

**Conclusion:**

Our study offers groundbreaking insights into the integration of metabolomics and genomics to reveal the pathogenesis of and therapeutic strategies for CKD. It underscores 48 metabolites as potential causal candidates, meriting further investigation.

**Supplementary Information:**

The online version contains supplementary material available at 10.1186/s12920-024-01918-3.

## Introduction

Chronic kidney disease (CKD), a medical condition characterized by sustained damage to kidney structure and function, is typically diagnosed when an individual’s estimated glomerular filtration rate (eGFR) falls below 60 mL/min per 1.73 m^2^, or if there are increased indicators of kidney damage persisting for at least three months [1]. This disease has a significant global impact, with an estimated prevalence rate between 10 and 15% worldwide. It directly contributes to mortality, morbidity, and comorbidity amongst various other complex characteristics [2]. By the year 2040, CKD is projected to rise as the fifth leading cause of death across the globe—marking it as one of the conditions with the largest anticipated increase in mortality rates [3]. Given that there currently exists no definitive cure for CKD, identifying its determinants becomes crucial for crafting effective primary prevention strategies.

Metabolites, the intermediate or end products of metabolic reactions, possess the capability to influence disease risk and serve as therapeutic intervention targets [4]. Accumulating evidence indicates a significant role for metabolic factors in CKD progression [5–8]. For instance, levels of 5-methoxytryptophan (5-MTP), a metabolite, decrease concomitant with CKD advancement. An overexpression of tryptophan hydroxylase-1 (TPH-1), an enzyme integral to 5-MTP synthesis, mitigates renal injury by attenuating inflammation and fibrosis in the renal system [9]. Moreover, another study delineated the association between plasma methylarginine concentration and the risk of CKD progression [10]. Various studies have underscored the consequential role of dysregulated kidney lipid metabolism in the progression of CKD [11–14]. But the mechanism of their connection remains to be elucidated. It’s crucial to note that these findings derive from observational studies. They may be subject to confounding bias or reverse causation due to the traditional study design [15]. Consequently, more comprehensive and systematic research is imperative to establish the causal relationship between CKD and metabolites definitively. Such investigations could provide novel insights into CKD mechanisms and potential targets for preventive and therapeutic strategies.

Mendelian randomization (MR) is a well-established method in genetic epidemiology that uses genetic variants as proxies for evaluating the causal relationships between exposures and disease outcomes [16]. Since genotypes are predetermined before the onset of disease and are unaffected by environmental factors, MR estimates are less prone to bias from confounding elements and reverse causation than traditional epidemiological studies. Hence, this study employed MR analysis to investigate the causal relationship between blood metabolites, CKD, and renal function. This approach offers valuable insights into the pathogenesis of CKD, paving the way for new predictive and therapeutic strategies for CKD.

## Methods

### Study design

**Fig. 1:**
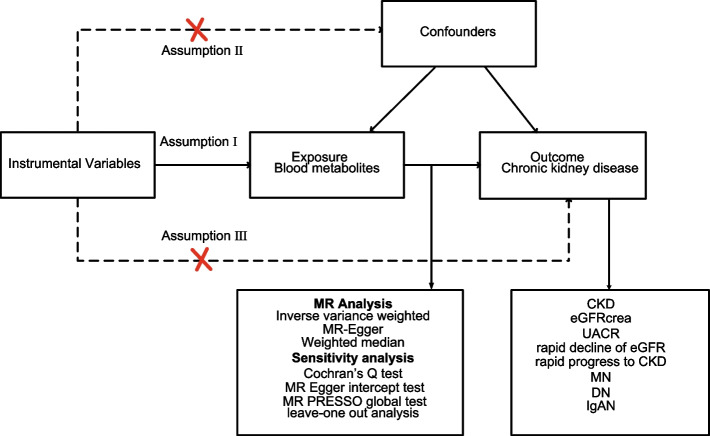
Overview of the current Mendelian randomization study. CKD, chronic kidney disease; eGFRcrea, creatinine-based estimated glomerular filtration rate; UACR, urine albumin to creatinine ratio; DN, diabetic nephropathy; IgAN, immunoglobulin A nephropathy; MN, membranous nephropathy

We conducted two-sample MR analyses using Genome-Wide Association Study (GWAS) summary statistics to estimate the causal effects of blood metabolites on CKD. The reliability of the results hinges on meeting three fundamental assumptions inherent in every MR analysis: (1) The instrumental variables (IVs) are strongly associated with exposure. (2) IVs are independent of unmeasured confounders. (3) The influence of IVs on outcomes is solely through their effect on exposure, rather than any other causal pathways [16]. Figure 1 presents a flowchart summarizing the entire procedure.

### Data source of exposure

Genetic instruments for metabolites were derived from a large-scale GWASinvolving 8,299 unrelated European subjects participating in the Canadian Longitudinal Study of Aging (CLSA). These participants have undergone genome-wide genotyping and had their circulating plasma metabolites measured [17]. Genotyping was carried out using the Affymetrix Axiom genotyping platform and imputed through the Trans-Omics for Precision Medicine (TOPMed) program [18]. Ancestry was determined by the CLSA group [19]. Prior to the GWAS, genotypic data underwent quality control, retaining single nucleotide polymorphisms (SNPs) with a minor allele frequency (MAF) above 0.1%, an imputation quality score greater than 0.3, and a missing rate less than 0.1%, resulting in approximately 15.4 million SNPs for the GWAS.

Metabolite levels were quantified by Metabolon, Inc. using an ultra-high performance liquid chromatography-tandem mass spectrometry (UPLC-MS/MS) platform, namely the Metabolon HD4 platform. During quality control, researchers ensured the accuracy and consistency of the metabolite data. Metabolites with less than 50% missing measurements in samples were retained (*N* = 1,091). Of these 1,091 tested plasma metabolites, 850 had known identities and were categorized into eight super-pathways: lipids, amino acids, xenobiotics, nucleotides, cofactors and vitamins, carbohydrates, peptides, and energy. The remaining 241 were labeled as “unknown” or “partially” characterized molecules. Metabolite levels underwent natural log transformation, outliers beyond 3 standard deviations were removed, and the data were standardized to have a mean of 0 and a standard deviation of 1.

In constructing metabolite ratios, researchers first identified 309 pairs of metabolites sharing enzymes or transport proteins using the Human Metabolome Database (HMDB) [20]. Metabolite ratios were calculated by dividing the batch-normalized measurement value of one metabolite by that of another in the same individual. These ratios were trimmed (retaining those within 3 standard deviations) and underwent inverse rank-normal transformation for statistical analysis(Supplementary Table 2 provides these pairs of metabolites and the HMDB evidence to support their pairing). Since many metabolites are substrates and products of enzymatic reactions, identifying genetic determinants of the ratio of substrate to product may provide information on biological processes that cannot be discerned when studying only single metabolites (Additional file 1: Supplementary Table S1-S3 provide these metabolites and metabolite ratios information).

### Data source of outcome

Given the long-term progression and multifactorial causes of CKD, we incorporated multiple endpoints in our analysis. These included CKD (primary outcome), creatinine-based eGFR (eGFRcrea), urine albumin to creatinine ratio (UACR), rapid progress to CKD (CKDi25) (defined as a decrease in eGFR ≥ 25% from baseline accompanied by progression from no CKD to CKD), rapid decline of eGFR (Rapid3) (an eGFR decrease of more than 3 mL/min/1.73 m^2^ per year), and specific renal diseases such as membranous nephropathy(MN), immunoglobulin A nephropathy(IgAN), and diabetic nephropathy(DN). MN, IgAN, and DN are common etiologies of CKD.

The GWAS summary statistics for CKD were derived from a meta-analysis conducted by the CKD Genetics Consortium (CKDGen), incorporating 23 European ancestry cohorts (*n* = 480,698; including 41,395 patients and 439,303 controls). All participants were of European ancestry, with a mean age of 54 y. All genetic associations were adjusted for sex, age, study site, genetic principal, components, relatedness, and other study-specific features [21]. The presence of CKD was defined by an eGFR less than 60 mL/min/1.73 m^2^. For the eGFRcrea, the GWAS summary statistics were obtained from a meta-analysis that comprised data from both the CKDGen Consortium and the UK Biobank, with a total of 1,201,909 participants [22]. In thr GWAS, eGFRcrea was winsorized at 15 or 200 ml/min/1.73 m^2^ and winsorized eGFRcrea values were log-transformed using a natural logarithm. For individuals 18 years or younger, the Schwartz formula was used to calculate eGFRcrea, while adults had their eGFRcrea calculated according to the CKD–EPI equation. A meta-analysis of GWAS data, encompassing 547,361 individuals of European descent, focused on the UACR. The UACR was measured in milligrams per gram, determined by dividing the urinary albumin concentration (mg/L) by the urinary creatinine concentration (mg/dL) and subsequently multiplying the quotient by 100 [23]. Summary statistics for Rapid3 (34,874 cases and 107,090 controls) and CKDi25 (19,901 cases and 175,244 controls) were sourced from a meta-analysis of 42 GWAS studies provided by the CKDGen Consortium and the UK Biobank [24]. Datasets for CKD, eGFR, UACR, Rapid3, and CKDi25 can be accessed at http://ckdgen.imbi.uni-freiburg.de/.

The GWAS summary statistics for MN were procured from the most recent study involving 2,150 cases and 5,829 controls of European ancestry. All cases used in this study were defined by a kidney biopsy diagnosis of idiopathic MN, with any suspected secondary cases due to drugs, malignancy, infection, or autoimmune disease being excluded [25]. Furthermore, the cohort underwent genotyping with high-density SNP arrays, and approximately 7 million common, high-quality genetic markers were imputed using the most up-to-date genome-wide sequence reference panel. The GWAS meta-analysis for IgAN integrated data from FinnGen, the UK Biobank, and the Biobank Japan, accounting for 477,784 individuals of European background (with 15,587 cases and 462,197 controls) and 175,359 individuals of East Asian descent (with 71 cases and 175,288 controls) [26]. Our analysis focused on the GWAS data from the European cohorts. The complete GWAS data pertaining to DN were derived from the latest published data in the FinnGen database (*n* = 312,650; including 4,111 patients and 308,539 controls) [27]. For detailed diagnostic criteria and inclusion procedures, please consult the original literature. Information about each dataset is available in Additional file 1: Supplement Table S4. The population involved in the exposure GWAS does not have sample overlap with the population used in the outcome GWAS.

### Selection of instrumental variables (IVs)

We first selected single-nucleotide polymorphisms (SNPs) associated with each metabolite at a significance threshold of *P* value < 1 × 10^–5^. This less stringent threshold is often chosen in MR analysis to capture greater variation for exposures when there are limited genome-wide significant SNPs available [28–30]. We subsequently carried out a clumping procedure to select independent SNPs using a linkage disequilibrium threshold of r^2 < 0.001 within a 10,000 kb window, referencing the European panel from the 1,000 Genomes Project. The next step included harmonizing the effect alleles of outcome-associated SNPs for consistency with those of exposure-associated SNPs. We removed SNPs with mismatched alleles, palindromic SNPs, and SNPs with missing values. To bolster the robustness of our findings, we applied Steiger filtering to eliminate SNPs exhibiting stronger correlations with outcomes rather than exposures [31]. Furthermore, to dismiss weak IVs, we calculated the F statistics for each IV and eliminated those with F-statistics < 10 [32]. The F statistic is computed using the formula F = R^2^(N-k-1)/[k(1-R^2^)], where R^2^ represents the proportion of variance in the exposure explained by the IVs, k is the number of IVs, N is the sample size. R^2^ was calculated by using formula: R^2^ = [2 × β^2^ × EAF × (1 − EAF)]/[2 × β^2^ × EAF × (1 − EAF) + 2 × *SE*^2^ × N × EAF × (1 − EAF)] [33]. β is the genetic effects on exposures, EAF is the effect allele frequency, SE is the standard error of the genetic effects, N is the sample size, In the formula, the numerator represents the variance in the exposure variable attributable to the SNP, while the denominator accounts for the total variance, which includes the variance explained by the SNP as well as the unexplained variance arising from environmental factors, random sampling error, or other genetic factors not taken into consideration. Consequently, the R^2^ value indicates the proportion of the variance in the exposure variable that is explained by the SNP relative to the total variance. A value closer to 1 suggests a stronger explanatory power of the SNP as an IV; conversely, a value approaching 0 indicates weaker explanatory capacity. The remaining SNPs, after these procedures, were ultimately used as IVs. If no IV is found in the outcome GWAS, we did not search for proxy SNPs.

### MR analysis

A two-sample MR analysis was conducted utilizing various methods, including the inverse-variance weighted (IVW), MR-Egger, and the weighted median. The IVW method served as our primary analytical tool. It amalgamates the Wald ratios of individual SNPs on the outcome to yield a consolidated causal estimate. Such a process yields the most accurate and unbiased estimates on the premise that all SNPs function as valid instrumental variables, or if any horizontal pleiotropy present is balanced [34]. When horizontal pleiotropy arises—when instrumental variables influence the outcome through exposure-unmediated pathways—MR-Egger regression comes into play [35]. The weighted median estimator can yield consistent results, providing that no less than 50% of the weight in the analysis originates from valid IVs [36].

Sensitivity analysis in MR studies plays an essential role in detecting potential genetic polymorphisms and the heterogeneity of MR estimates. The heterogeneity among the instruments was gauged using Cochran’s Q test. We utilized the MR-Egger intercept test [35] and MR Pleiotropy RESidual Sum and Outlier (MR-PRESSO) global test [37] to examine potential horizontal pleiotropy effects and assess the presence of directional pleiotropy. A leave-one-out analysis was carried out to identify and exclude potential outliers that might independently influence the observed causal relationship.

To account for multiple testing, we employed the Bonferroni correction’s multiple-testing-adjusted threshold, correcting for the quantity of exposures tested for each metabolite. The significance of the causal feature was set at *P* < 3.571 × 10^−5^ (0.05/1400). Bonferroni’s correction offers a straightforward way to control the type I error rate by dividing the critical significance level by the number of tests performed. Nonetheless, it should be noted that correction procedures tend to become overly conservative when a high volume of tests is conducted [38]. Hence, we identified serum metabolites as nominal significant features if *P* < 0.05 in all three MR analyses and with no heterogeneity or pleiotropy was detected. Statistical evaluations were executed in R4.2.3 software, and MR analyses were carried out using the TwoSampleMR package.

## Results

### IVs for exposures

Some metabolites did not find SNPs that met the criteria, ultimately obtaining 1352 metabolites’ IVs (Additional file 1: Supplementary Table S5). The number of SNPs used as IVs ranged from 12–46 (median, 26) for the metabolites and metabolite ratios. The median F statistic was 21.2 (ranged from 19.5 to 1064.9) for metabolites and metabolite ratios, an F-statistic > 10 is considered sufficiently informative for MR analyses.

### Association of serum metabolites with CKD

**Fig. 2:**
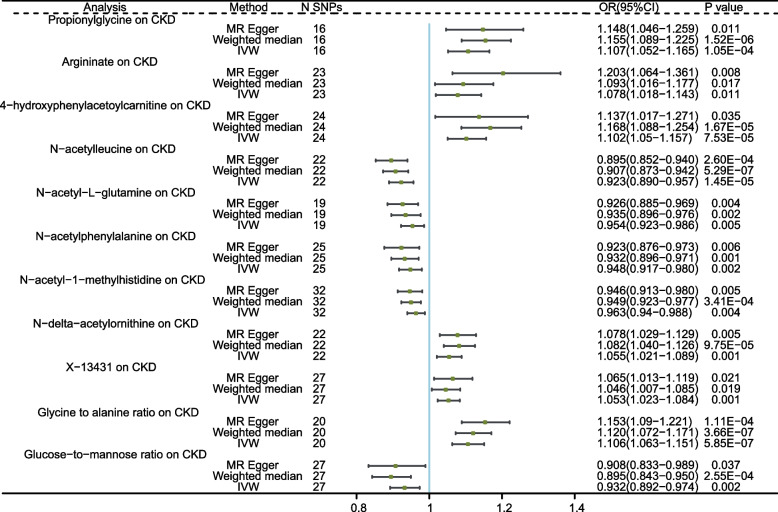
MR analysis of the causality of serum metabolites on CKD. CKD, chronic kidney disease; IVW, inverse variance weighted; SNP, single nucleotide polymorphism; OR, odds ratio; CI, confidence interval

Results from the Bonferroni-corrected test revealed that N-acetylleucine level retains a strong causal relationship with lower risk of CKD (OR = 0.923, 95%CI: 0.89–0.957, *P*_IVW_ = 1.450 × 10^–5^), whereas Glycine to alanine ratio retains a strong causal relationship with higher risk of CKD (OR = 1.106, 95%CI: 1.063–1.151, *P*_IVW_ = 5.850 × 10^–7^). Additionally, 9 genetically predicted serum metabolites showed significant associations with CKD risk at the nominal significance level of 0.05 (*P* < 0.05 for all three MR analyses). The levels of genetically predicted Propionylglycine, Argininate, 4-hydroxyphenylacetoylcarnitine, N-delta-acetylornithine, and X-13431 were associated with a higher risk of CKD. Conversely, the levels of N-acetyl-L-glutamine, N-acetylphenylalanine, N-acetyl-1-methylhistidine and Glucose-to-mannose ratio were associated with a lower risk (as shown in Fig. 2). Information on the IVs used in the MR analysis can be found in Additional file 1: Supplementary Table S6, and the results of the MR analysis for all metabolites in relation to CKD are presented in Supplementary Table S7. The results from Cochrane’s Q test (Additional file 1: Supplement Table S8) showed that no obvious heterogeneity was found in the selected SNPs (*P* > 0.05). Furthermore, the MR-Egger and MR-PRESSO tests (Additional file 1: Supplement Table S8) showed that there is no horizontal pleiotropy (*P* > 0.05). The scatter plots for the causal relationship between metabolites and CKD was presented in Additional file 2: Supplementary Figure S1. The results of leave-one-out sensitivity were shown in Additional file 2: Supplementary Figure S2.

### Association of serum metabolites with eGFRcrea

**Fig. 3:**
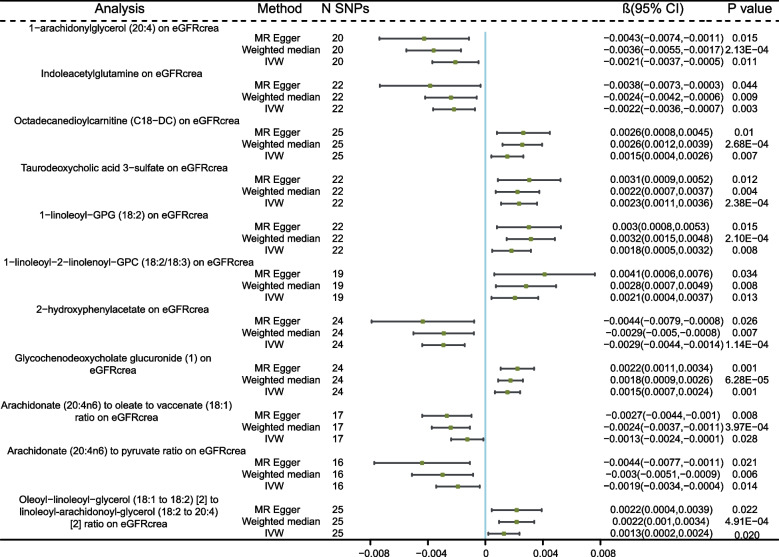
MR analysis of the causality of serum metabolites on eGFRcrea. eGFRcrea, creatinine-based estimated glomerular filtration rate; IVW, inverse variance weighted; SNP, single nucleotide polymorphism; β, effect size; CI, confidence interval

Despite the Bonferroni correction yielding no significant causal associations between serum metabolites and eGFRcrea, 11 genetically predicted serum metabolites showed significant associations with eGFRcrea at the nominal significance level of 0.05 (*P* < 0.05 for all three MR analyses). A higher genetically predicted 1-arachidonylglycerol (20:4), Indoleacetylglutamine, 2-hydroxyphenylacetate, Arachidonate (20:4n6) to oleate to vaccenate (18:1) ratio, and Arachidonate (20:4n6) to pyruvate ratio were associated with a decrease in eGFR. While Octadecanedioylcarnitine (C18-DC),Taurodeoxycholic acid 3-sulfate, 1-linoleoyl-GPG (18:2), 1-linoleoyl-2-linolenoyl-GPC (18:2/18:3), Glycochenodeoxycholate glucuronide (1), and Oleoyl-linoleoyl-glycerol (18:1 to 18:2) [2] to linoleoyl-arachidonoyl-glycerol (18:2 to 20:4) [2] ratio were associated with an increase in eGFR (Fig. 3). MR-Egger and MR-PRESSO tests (Supplement Table S8) showed that there is no horizontal pleiotropy (*P* > 0.05). Furthermore, no obvious heterogeneity was found according to results from Cochrane’s Q test (Supplement Table S8) (*P* > 0.05). Information on the IVs used in the MR analysis can be found in Additional file 1: Supplementary Table S9, and the results of the MR analysis for all metabolites in relation to eGFRcrea are presented in Supplementary Table S10. The scatter plots for the causal relationship between metabolites and eGFRcrea were presented in Additional file 2: Supplementary Figure S3. The results of leave-one-out sensitivity were shown in Additional file 2: Supplementary Figure S4.

### Association of serum metabolites with UACR

**Fig. 4:**
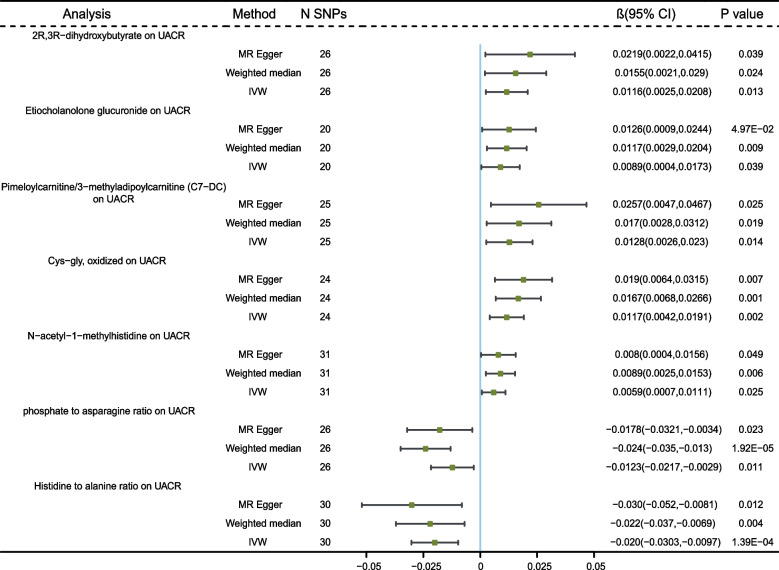
MR analysis of the causality of serum metabolites on UACR. UACR, urine albumin to creatinine ratio; IVW, inverse variance weighted; SNP, single nucleotide polymorphism; β, effect size; CI, confidence interval

The effects of genetically predicted serum metabolites on the level of UACR are presented in Fig. 4. No serum metabolite remained significant after the Bonferroni correction. But 7 genetically predicted serum metabolites showed significant associations with UACR at the nominal significance level of 0.05. A higher genetically predicted 2R,3R-dihydroxybutyrate, Etiocholanolone glucuronide, Pimeloylcarnitine/3-methyladipoylcarnitine (C7-DC), Cys-gly oxidized, N-acetyl-1-methylhistidine were associated with an increase in UCAR, while phosphate to asparagine ratio and Histidine to alanine ratio were associated with the decrease in UACR. MR-Egger and MR-PRESSO tests did not show evidence of horizontal pleiotropy. We found no evidence of heterogeneity in the course of our analysis(Additional file 1: Supplement Table S8). The IVs used in the MR analysis and all MR results were shown in Additional file 1: Supplementary Tables S11-12. The scatter plots for the causal relationship between metabolites and UACR were presented in Additional file 2: Supplementary Figure S5. The results of leave-one-out sensitivity were shown in Additional file 2: Supplementary Figure S6.

### Association of serum metabolites with CKDi25

**Fig. 5:**
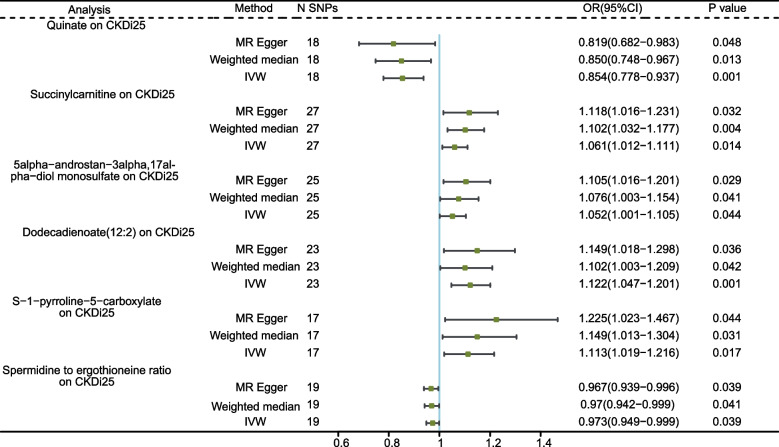
MR analysis of the causality of serum metabolites on CKDi25. CKDi25, defined as a decrease in eGFR ≥ 25% from baseline accompanied by progression from no CKD to CKD; IVW, inverse variance weighted; SNP, single nucleotide polymorphism; OR, odds ratio; CI, confidence interval

The effects of genetically predicted serum metabolites on the CKDi25 are presented in Fig. 5. No serum metabolite remained significant after the Bonferroni correction. But 6 genetically predicted serum metabolites showed significant associations with CKDi25 at the nominal significance level of 0.05. The direction of these genetically predicted serum metabolites was consistent across the three MR methods tested. And *P* < 0.05 for all three MR analyses. A higher genetically predicted Quinate levels and Spermidine to ergothioneine ratio were associated with a lower risk of CKDi25, while the Succinylcarnitine, 5alpha-androstan-3alpha,17alpha-diol monosulfate, Dodecadienoate (12:2) and S-1-pyrroline-5-carboxylate levels were associated with a higher risk of CKDi25. Additional file 1: Supplementary Tables S13-14 respectively provided the IVs used in the MR analysis and the MR results. MR-Egger, MR-PRESSO tests and Cochrane’s Q test did not find horizontal pleiotropy, outliers and heterogeneity (Additional file 1: Supplement Table S8). The scatter plots and the results of leave-one-out sensitivity were shown in Additional file 2: Supplementary Figure S7-S8.

### Association of serum metabolites with Rapid3

**Fig. 6:**
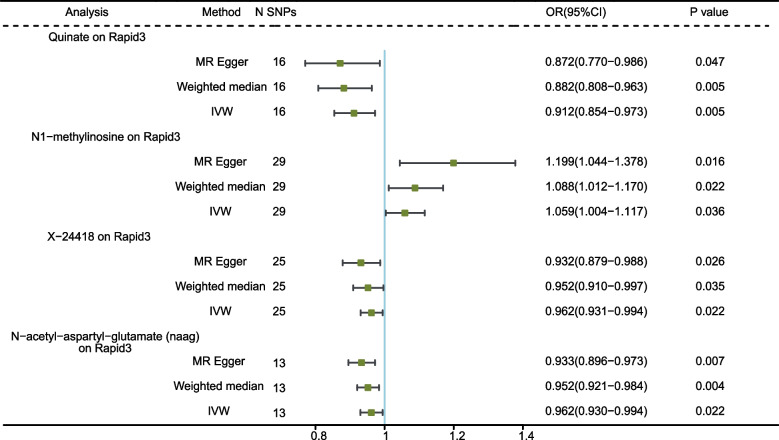
MR analysis of the causality of serum metabolites on Rapid3. Rapid3, rapid decline of eGFR, an eGFR decrease of more than 3 mL/min/1.73 m^2^ per year; IVW, inverse variance weighted; SNP, single nucleotide polymorphism; OR, odds ratio; CI, confidence interval

Four genetically predicted serum metabolites showed significant associations with Rapid3. A higher genetically predicted Quinate, X-24418, and N-acetyl-aspartyl-glutamate (naag) levels were associated with a lower risk of Rapid3, while the N1-methylinosine level was associated with a higher risk of Rapid3 (Fig. 6). No significant heterogeneity and horizontal pleiotropy were found according to Cochrane’s Q, MR-Egger, and MR-PRESSO tests (Additional file 1: Supplement Table S8). The IVs utilized in the MR analysis and the corresponding MR results were respectively detailed in Supplementary Tables S15 and 16. The scatter plots and the results of leave-one-out sensitivity were shown in Additional file 2: Supplementary Figure S9-S10.

### Association of serum metabolites with MN

The effects of genetically predicted serum metabolites on MN are presented in Supplement Figure S11. Hydroxy-N6,N6,N6-trimethyllysine, N-succinyl-phenylalanine, X-17685 levels were associated with a reduced risk of MN. The IVs used in the MR analysis and all MR results were shown in Additional file 1: Supplementary Tables S17-18. No significant heterogeneity and horizontal pleiotropy were found according to Cochrane’s Q, MR-Egger, and MR-PRESSO tests (Additional file 1: Supplement Table S8). The scatter plots and the results of leave-one-out sensitivity were shown in Additional file 2: Supplement Figures S12-S13.

### Association of serum metabolites with DN

The effects of genetically predicted serum metabolites on DN are presented in Supplement Figure S14. A higher genetically predicted Phosphate to threonine ratio, Aspartate to N-acetylglucosamine to N-acetylgalactosamine ratio, Caffeine to paraxanthine ratio and Paraxanthine to 5-acetylamino-6-formylamino-3-methyluracil ratio were associated with a lower risk of DN, while the 2-aminophenol sulfate and N-acetyl-isoputreanine levels were associated with a higher risk of DN. No evidence of heterogeneity or horizontal pleiotropy was found during our analysis (Additional file 1: Supplementary Table S8). Information on the IVs used in the MR analysis can be found in Additional file 1: Supplementary Table S19, and the results of the MR analysis for all metabolites in relation to DN are presented in Supplementary Table S20. Scatter plots and the results of leave-one-out sensitivity analysis were provided in Additional file 2: Supplement Figures S15 and S16, respectively.

### Association of serum metabolites with IgAN

The effects of genetically predicted serum metabolites on IgAN are presented in Supplement Figure S17. Higher genetically predicted levels of X-11858 and N-acetyl-1-methylhistidine were associated with an increased risk of IgAN. Conversely, 3-(4-hydroxyphenyl)lactate level was associated with a reduced risk of IgAN. The results of Cochrane’s Q, MR-Egger, and MR-PRESSO tests were presented in Additional file 1: Supplementary Table S8. The IVs utilized in the MR analysis and the corresponding MR results were respectively detailed in Additional file 1: Supplementary Tables S21 and 22. The scatter plots and the results of leave-one-out sensitivity were shown in Additional file 2: Supplementary Figures S18-S19.

### Shared causal metabolites

**Fig. 7:**
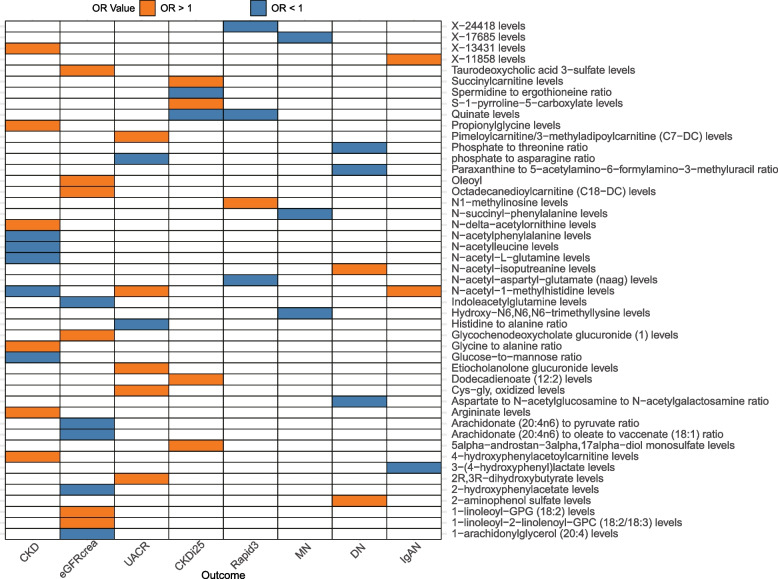
Heatmap plot of causal associations between circulating metabolites and outcomes. The x-axis shows the categories of 7 outcomes. The y-axis presents significant metabolites. CKD, hronic kidney disease; eGFRcrea, creatinine- based estimated glomerular filtration rate; UACR, urine albumin to creatinine ratio; CKDi25, defined as the decrease of eGFR ≥ 25% of baseline accompanied by the progression from no CKD to CKD; Rapid3: the eGFR decreases by more than 3 mL/min/1.73 m^2^ per year; MN, membranous nephropathy; IgAN, immunoglobulin A nephropathy; DN, diabetic nephropathy; OR, Odds Ratio;OleoyO, leoyl − linoleoyl − glycerol (18:1 to 18:2) [2] to linoleoyl − arachidonoyl − glycerol (18:2 to 20:4) [2] ratio; OR, odds ratio

Figure 7 illustrates the associations between metabolites and various renal outcomes. It can be observed that Quinate is associated with 2 renal outcomes: CKDi25 and Rapid3, both showing a consistent direction of effect (OR < 1). Levels of N-acetyl-1-methylhistidine are associated with 3 renal outcomes, specifically with CKD(OR: 0.963), with UACR(β: 0.0059), and with IgAN(OR: 1.037).

## Discussion

In the present study, we performed an exhaustive two-sample MR analysis, employing GWAS summary statistics, to explore potential associations between 1,400 serum metabolites in humans and eight CKD phenotypes (CKD, eGFRcrea, UACR, Rapid3, CKDi25, MN, IgAN, and DN). The causal relationships inferred exhibited robustness. A total of 48 metabolites demonstrating associations with CKD phenotypes were identified, with two of them showing a potent causal relationship. Specifically, a strong association was observed between N-acetylleucine levels and a reduced risk of CKD (OR = 0.923, 95% CI: 0.89–0.957, *P*_IVW_ = 1.450 × 10^–5^), while an elevated risk of CKD was strongly linked to the glycine to alanine ratio (OR = 1.106, 95% CI: 1.063–1.151, *P*_IVW_ = 5.850 × 10^–7^). To our knowledge, this represents the first comprehensive MR study integrating metabolomics with genomics to assess the causal relationship between metabolites and CKD.

N-acetylleucine (NALL) is the N-acetyl derivative of L-leucine and plays a role as a metabolite. A metabolomics study on patients undergoing hemodialysis identified 397 named solutes by comparing samples from patients with those from healthy controls. Out of these solutes, 120 were identified as uremic solutes. These solutes include multiple N-Acetylated standard amino acids [39]. Another study, which included 3,906 individuals from the Hispanic/Latino population, identified multiple metabolites associated with eGFR, including N-acetylcarnosine, N-formylmethionine, N-acetylalanine, and N-acetylthreonine. Notably, about 3% (126 individuals) of the population included in this study had CKD(< 60 mL/min/1.73 m^2^), with the majority of these individuals having mild CKD (stage G3a) [40]. However, in our MR analysis, we only found an association between NALL and a reduced risk of CKD, with the association between NALL and eGFR not being replicated (Additional file 1: Supplementary Table S10). This may be due to the fact that the population included in the GWAS for eGFR had higher eGFR levels compared to the population included in the GWAS for CKD [22]. At the same time, certain observational studies, clinical trials, and cellular experiments provide partial support for NALL’s beneficial impact on disease treatment. In observational studies, acetyl-leucine has been shown to exert symptomatic and long-term, disease-modifying effects in patients suffering from GM2 gangliosidoses and other lysosomal disorders such as Niemann-Pick disease type C (NPC) [41–45]. A recent parallel, multinational, phase IIb clinical trial involving NALL for NPC demonstrated statistically significant (primary and secondary endpoints) and clinically meaningful improvement in symptoms, functioning, and quality of life in both children and adults with NPC [41]. Moreover, a phase IIb, multinational, open-label, rater-blinded study revealed that treatment with NALL was associated with statistically significant and clinically relevant improvements in functioning and quality of life in patients with GM2 gangliosidosis [46]. NALL was deemed safe and well-tolerated, with no serious adverse reactions reported. NALL exhibits multiple biological effects, including the activation of brain glucose metabolism in the cerebellum [47, 48], regulation of lipid and cholesterol accumulation and lytic enzyme volume [45, 49], reduction of neuroinflammation [50], and promotion of normal neuronal membrane potential [51]. These functions facilitate neuronal functional recovery, thereby enhancing brain health.

Our MR analysis indicates that the glycine to alanine ratio is associated with an increased risk of CKD (OR = 1.106, 95% CI: 1.063–1.151, *P*_IVW_ = 5.850 × 10^–7^). It is known that results for ratios might be driven by components of that ratio. The MR results reveal that the alanine level is not associated with CKD (*P* > 0.05), but the glycine level is related to an increased risk of CKD, with results from IVW (OR = 1.066, 95%CI: 1.026–1.107, *P* = 9.66 × 10^–4^), MR Egger (OR = 1.129, 95%CI: 1.08–1.18, *P* = 2.63 × 10^–5^), and Weighted median (OR = 1.091, 95%CI: 1.052–1.131,*P* = 2.16 × 10^–6^) (Additional file 1: Supplementary Table S7). The MR-Egger method indicates the presence of pleiotropy(*P* = 0.002). Even though the results of the MR-Egger method take into account a certain degree of pleiotropy, the results at this point are still unstable. Therefore, glycine was not included in our results. The comprehensive analysis indicates that the correlation between the glycine to alanine ratio and the risk of CKD may be driven by glycine. The results of the glycine to alanine ratio also support the association between increased glycine levels and a higher risk of CKD. The ratio itself is also meaningful, as it can support the metabolite from another perspective. Glycine, a non-essential amino acid, plays numerous roles and has various effects. It is obtained from dietary intake and endogenous synthesis, primarily in the liver and kidneys. Yet, the role of glycine in renal pathology remains to be fully elucidated. In rats with streptozotocin-induced diabetes, glycine has been shown to reduce renal oxidative stress by inhibiting Nox4 expression, thereby mitigating diabetic renal damage [52]. Urinary glycine may hold diagnostic and prognostic value for IgA nephropathy (IgAN), suggesting that urinary glycine may serve as a protective biomarker for IgAN [53]. Furthermore, glycine has demonstrated the ability to alleviate nephrotoxicity induced by cyclosporine, cisplatin, cadmium, and lead [54–59]. Regarding renal injury caused by ischemia–reperfusion (I/R), some studies have indicated that glycine can mitigate ischemia–reperfusion injury in the kidney in vivo [60, 61]. Conversely, other studies have found no protective effect or even an exacerbation of renal injury by glycine in I/R-induced kidney injury [62, 63]. In vitro experiments suggest that glycine-conjugated metabolites play a significant role in renal injury and hypertension, with a reduction in glycine-conjugated metabolites leading to decreased renal fibrosis [64]. Another study revealed that intramitochondrial aggregates composed of mutated Glycine Amidinotransferase (GATM) protein are causative for an autosomal dominant form of renal Fanconi syndrome and CKD. The emergence of these aggregates was accompanied by an increase in the production of reactive oxygen species (ROS), inflammatory signals, cell death, and renal fibrosis [65]. A link between genetic variation in the GATM gene and plasma creatinine levels was previously suggested by GWAS [66, 67].

It is important to note that there is a potential for false negatives in the Bonferroni-corrected test. Our study identified several metabolites—including Propionylglycine, 4-hydroxyphenylacetoylcarnitine, N-delta-acetylornithine, X-13431, N-acetyl-L-glutamine, N-acetylphenylalanine, N-acetyl-1-methylhistidine and Glucose-to-mannose ratio—that are closely associated with CKD. Specifically, N-acetyl-1-methylhistidine is associated with CKD, UACR, and IgAN. Quinate is associated with CKDi25 and Rapid3. However, these correlations were not maintained after the Bonferroni correction. It is worth mentioning that the three MR methods provided consistent results for the analysis of these metabolites (*P* < 0.05 for all three MR analyses). Therefore, these metabolites can be considered as potential causal factors and warrant further investigation. They should not be disregarded based on the results of the Bonferroni correction alone. The same caveat applies to other outcomes. This is a key point that we wish to emphasize.

Our study was subject to several limitations. First, for numerous metabolites, only a limited number of genome-wide significant SNPs were available for MR analysis. To circumvent this issue, we adopted a relatively relaxed significance threshold for IVs selection, as recommended by prior studies [28–30, 68]. Second, our data were sourced from European populations, which may limit the generalizability of our findings to a broader demographic, although this approach does mitigate the risk of population structure bias. Third, while our study suggests associations between certain metabolites and CKD risk, it mainly offers predictive insights without empirical validation. The causal associations and underlying molecular mechanisms require more comprehensive investigation and confirmation in future research. Fourth, the semi-quantitative approach to metabolite measurement, coupled with data collection at a single time point, presents a limitation. This approach may not fully capture the temporal dynamics of metabolite levels and their potential correlation with the progression of chronic kidney disease. Future studies would benefit from incorporating quantitative methodologies and longitudinal data collection to enhance the understanding of these relationships over time. Lastly, metabolite levels are known to fluctuate across different cell types and tissues. However, in this study, we could only evaluate the impact of metabolites measured in blood on CKD, and were unable to assess the relevance of metabolite levels in more biologically pertinent tissues, such as the kidney.

## Conclusions

In conclusion, our study identified several metabolites with potential causal roles in CKD and renal function. Notably, we underscored the Glycine to Alanine ratio as a promising risk-increasing metabolite, and N-acetylleucine as a protective candidate in CKD, both of which are backed by strong MR evidence. Our research offers innovative insights into the integration of metabolomics and genomics, shedding light on the pathogenesis of CKD and potential therapeutic strategies.

### Supplementary Information

Supplementary Material.

Supplementary Material.

## Data Availability

Publicly available datasets were analyzed in this study. This data can be found here: (http://ckdgen.imbi.uni-freiburg.de/), (https://www.finngen.fi/) and (https://www.ebi.ac.uk/gwas/).

## Abbreviations

Ala: Alanine
CKD: Chronic kidney disease
CKDi25: Rapid progress to CKD
CLSA: Canadian Longitudinal Study of Aging
DN: Diabetic nephropathy
eGFR: Estimated glomerular filtration rate
eGFRcrea: Creatinine-based eGFR
GATM: Glycine amidinotransferase
GWAS: Genome-wide association study
I/R: Ischemia–reperfusion
IgAN: Immunoglobulin A nephropathy
IVs: Instrumental variables
IVW: Inverse variance weighted
MN: Membranous nephropathy
MR: Mendelian randomization
MR-PRESSO: MR Pleiotropy RESidual Sum and Outlier
NALL: N-acetylleucine
Rapid3: Rapid decline of eGFR
ROS: Reactive oxygen species
SNPs: Single-nucleotide polymorphisms
TPH-1: Tryptophan hydroxylase-1
UACR: Urine albumin to creatinine ratio
5-MTP: 5-Methoxytryptophan
