# Supplementary material for: Blood metabolites and chronic kidney disease: a Mendelian randomization study
Source: BMC Med Genomics. 2024 May 28;17:147. doi: 10.1186/s12920-024-01918-3 (PMC11131213; doi:10.1186/s12920-024-01918-3)
Supplement: Supplementary file 2 — Supplementary Material. [file 12920_2024_1918_MOESM2_ESM.docx]

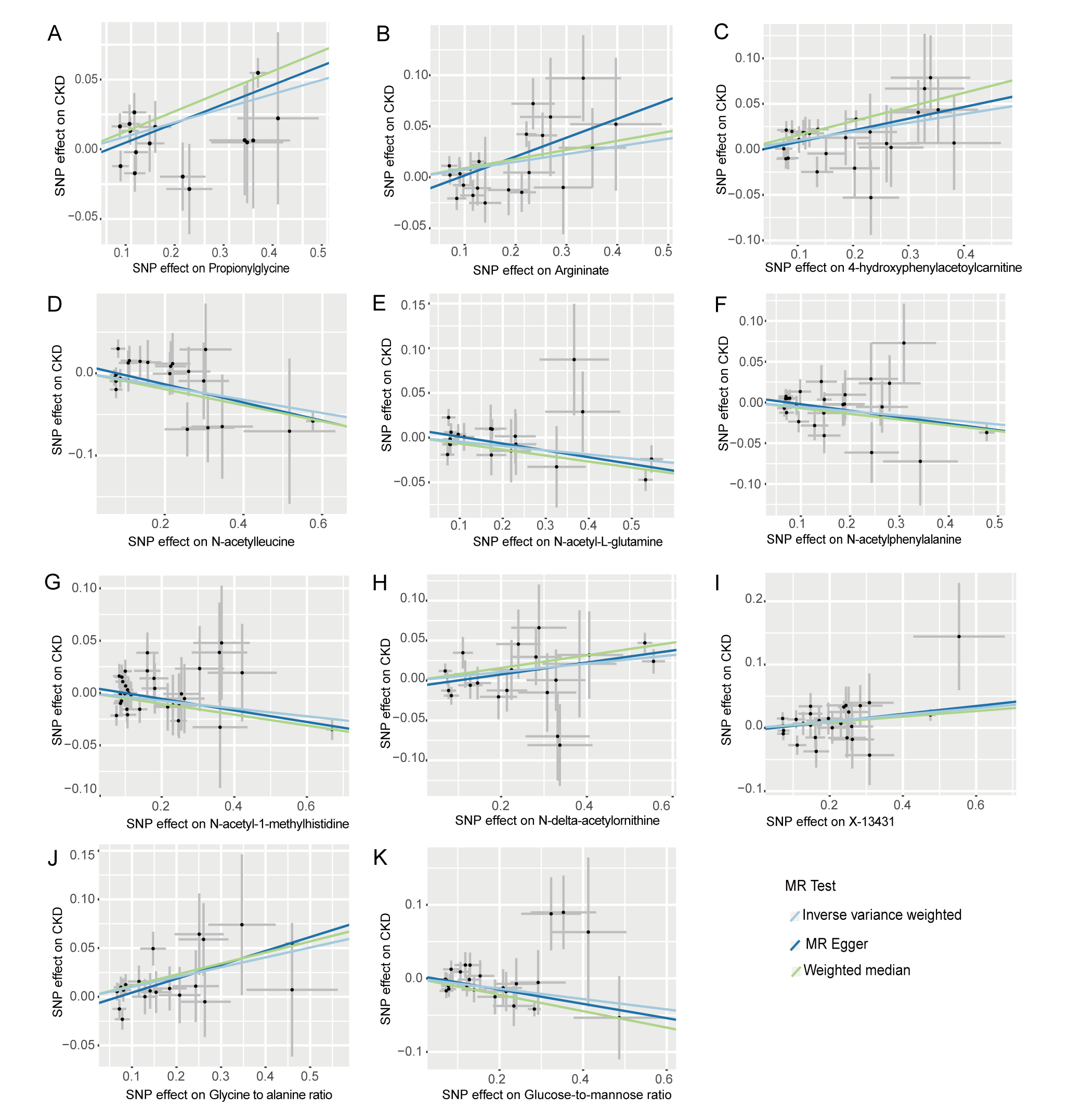


Supplement Figure S1. Scatter plot of the MR analysis results for the effect of the serum metabolites on CKD. CKD, chronic kidney disease; MR, Mendelian randomization; SNP, single nucleotide polymorphism.


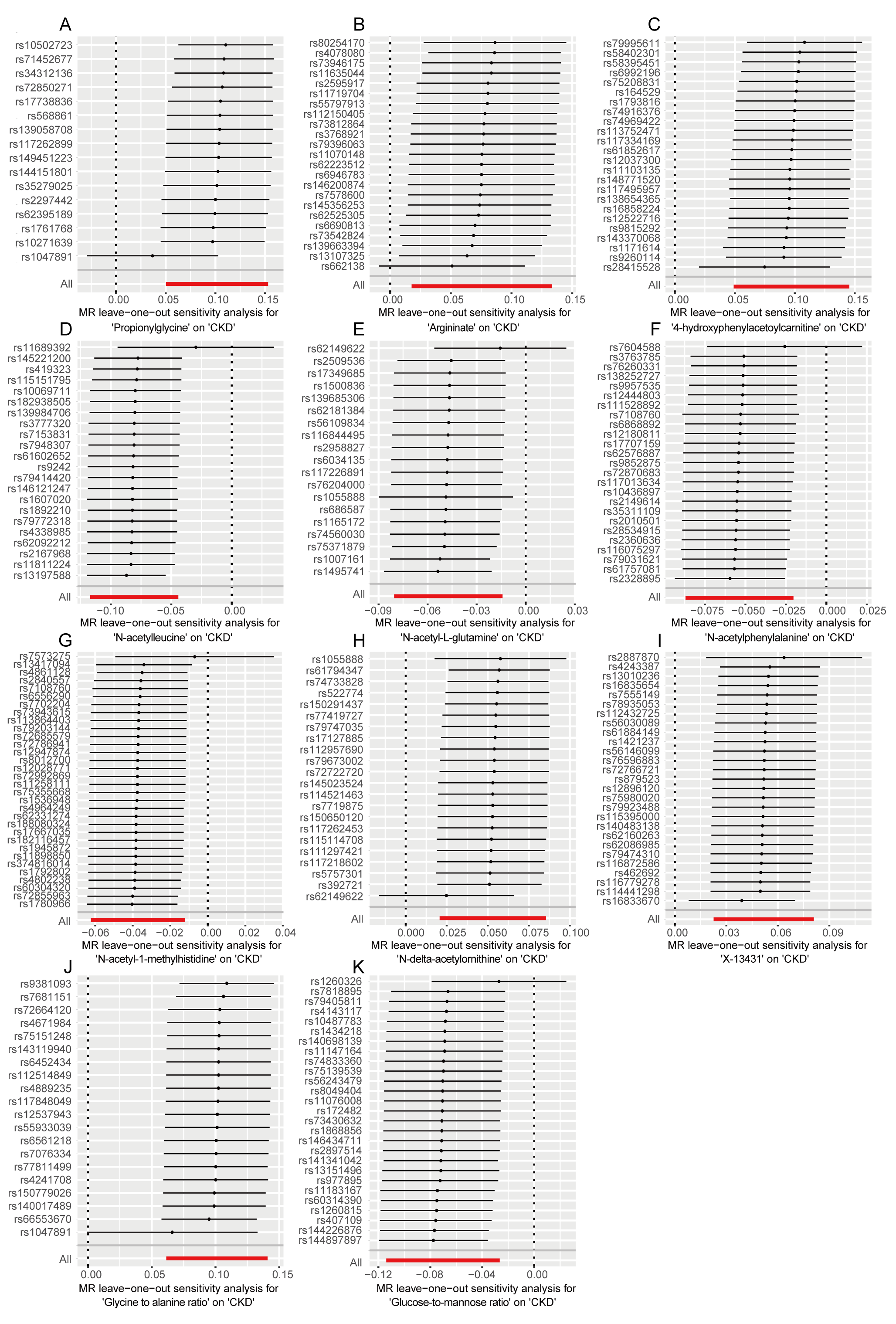


Supplement Figure S2. MR leave-one-out sensitivity analysis of serum metabolites -associated SNPs with risk of CKD.


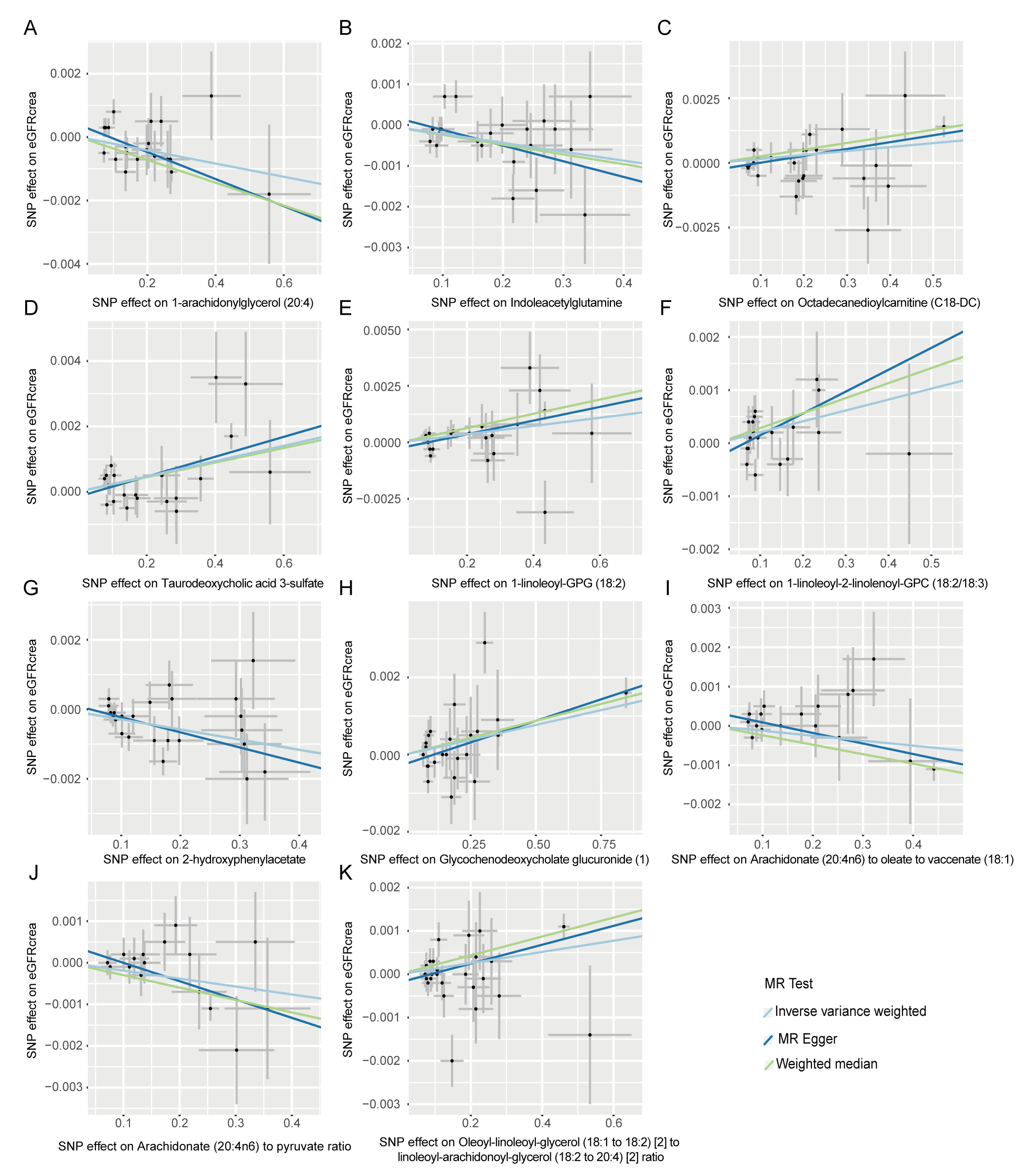


Supplement Figure S3. Scatter plot of the MR analysis results for the effect of the serum metabolites on eGFRcrea.


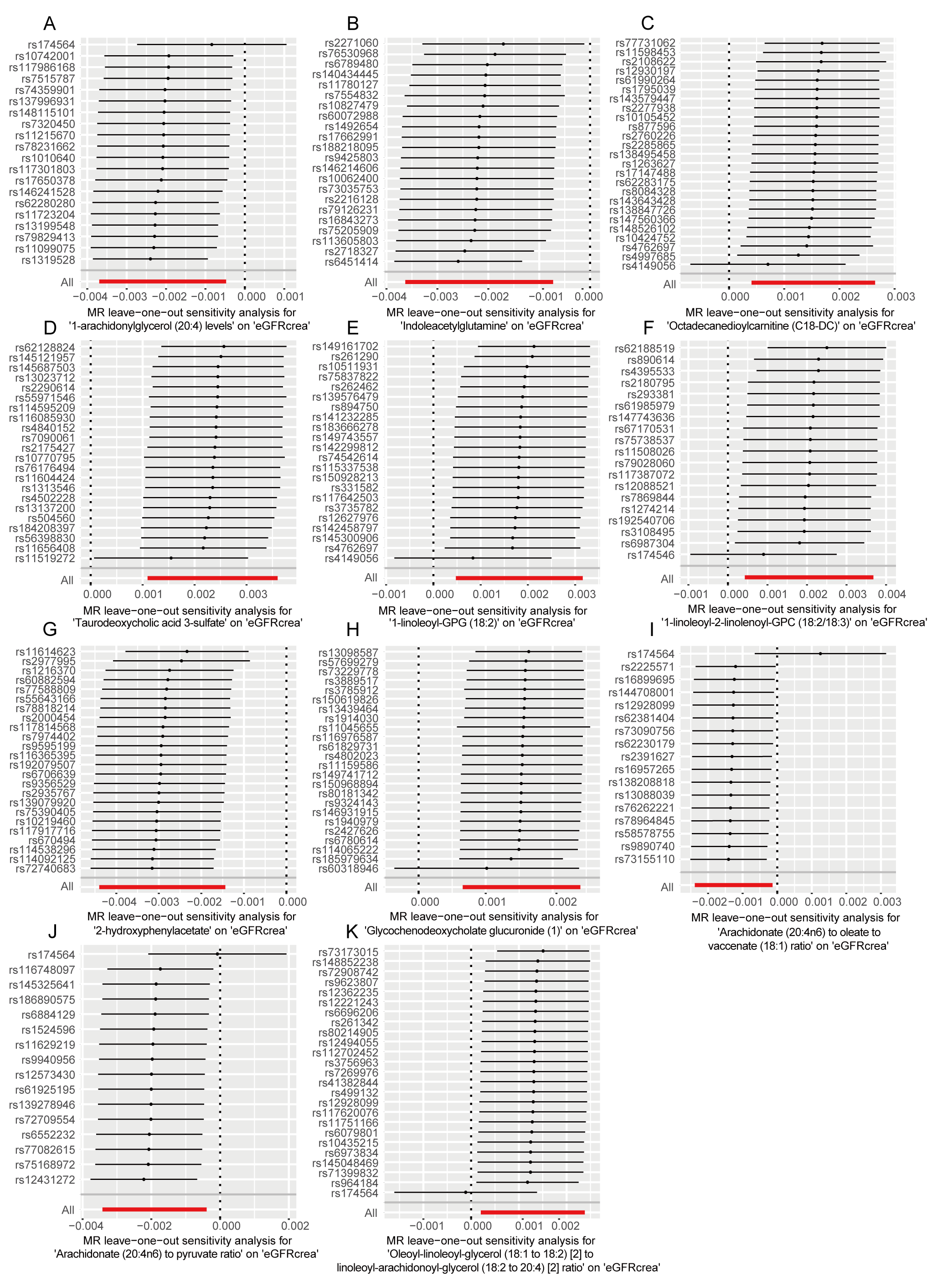


Supplement Figure S4. MR leave-one-out sensitivity analysis of serum metabolites -associated SNPs with risk of eGFRcrea.


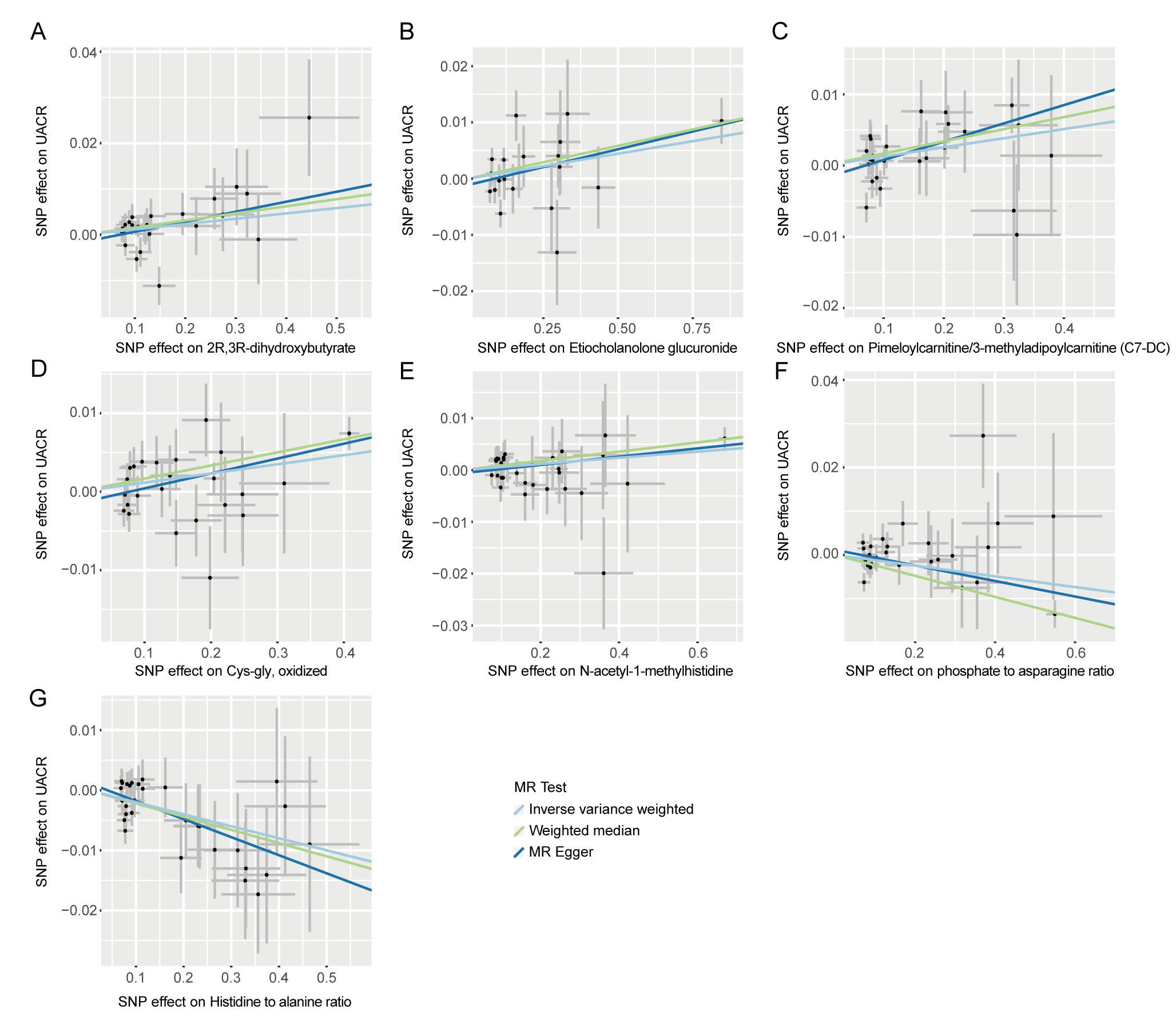


Supplement Figure S5. Scatter plot of the MR analysis results for the effect of the serum metabolites on UACR.


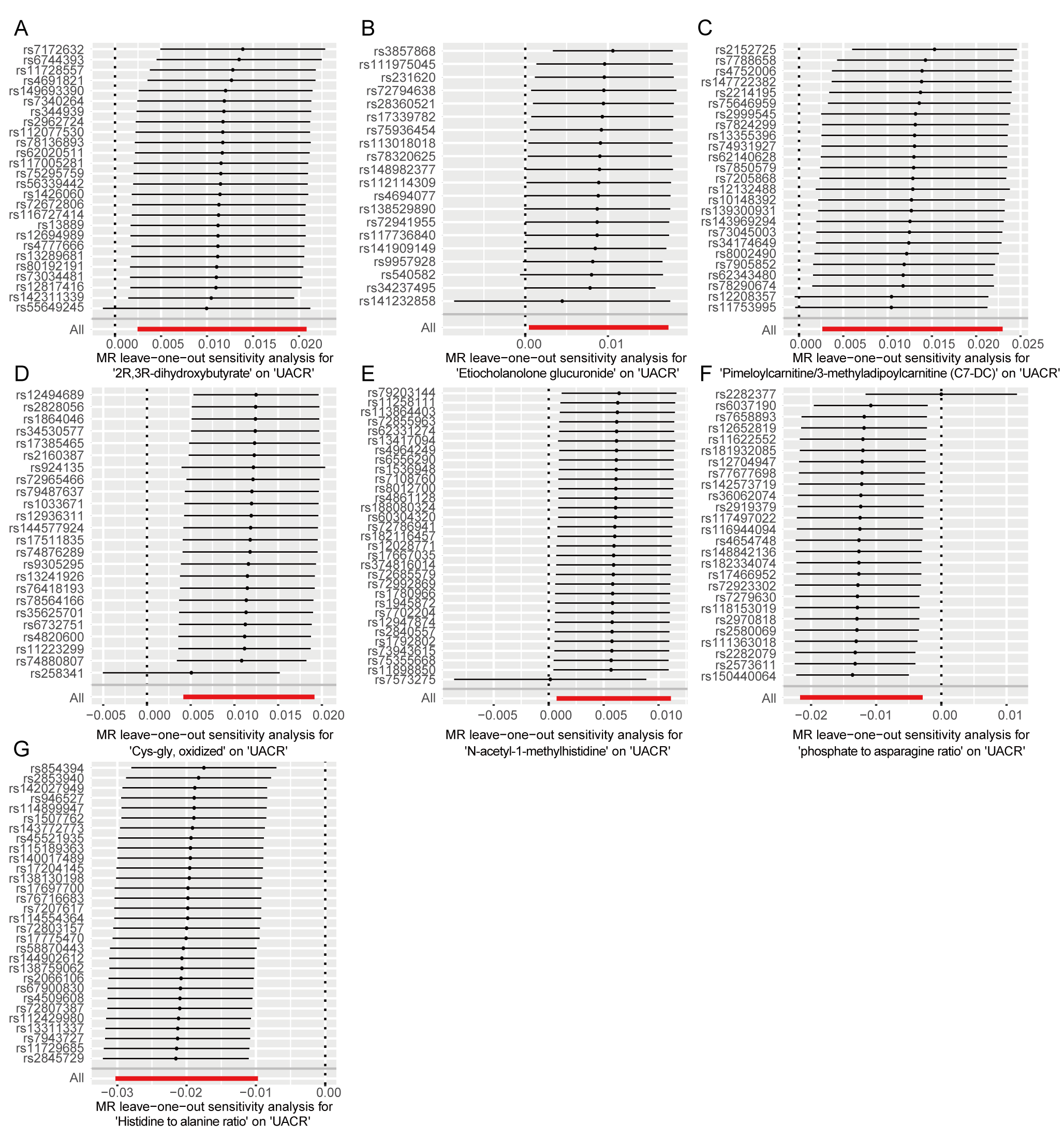


Supplement Figure S6. MR leave-one-out sensitivity analysis of serum metabolites -associated SNPs with risk of UACR.


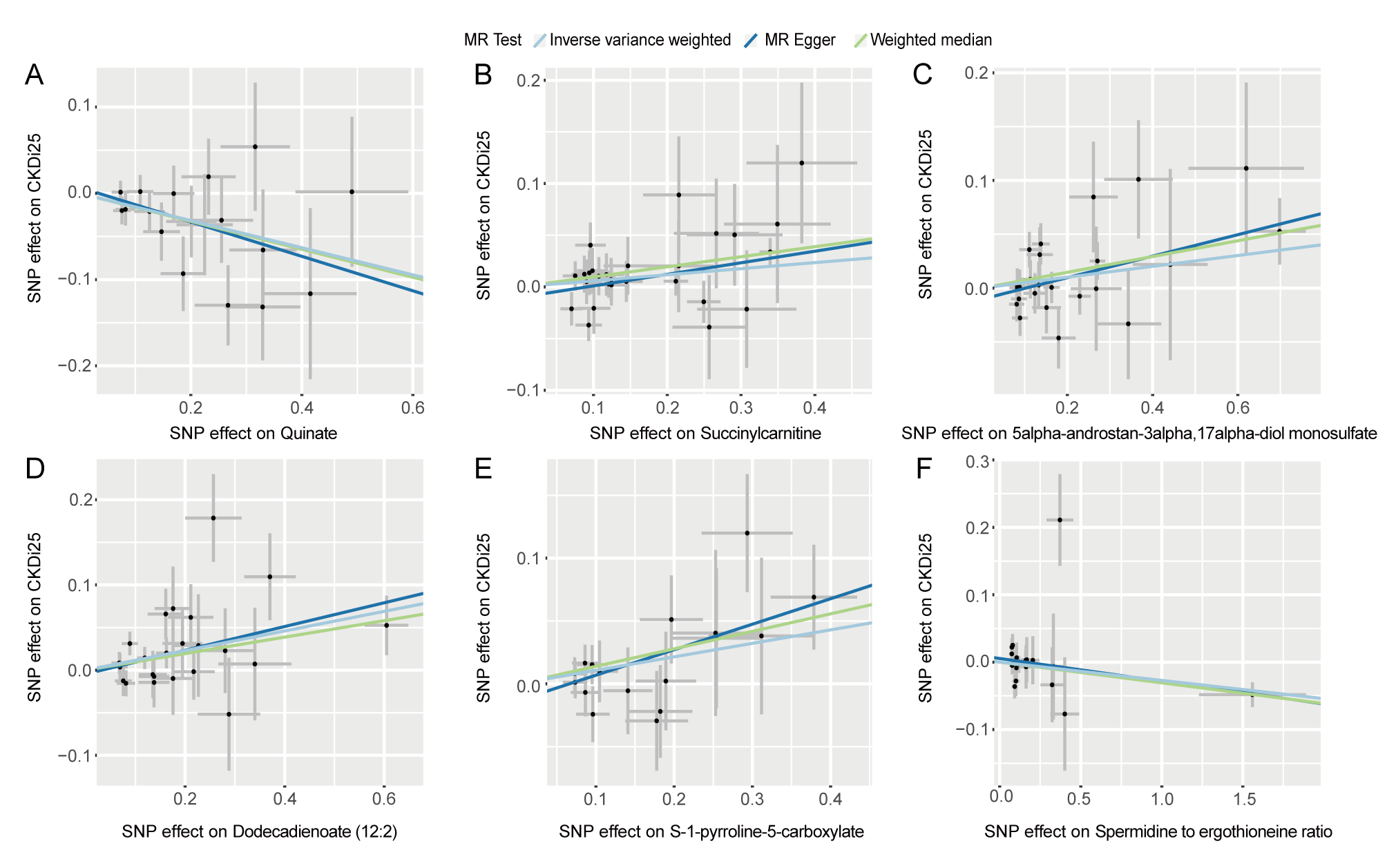


Supplement Figure S7. Scatter plot of the MR analysis results for the effect of the serum metabolites on CKDi25.


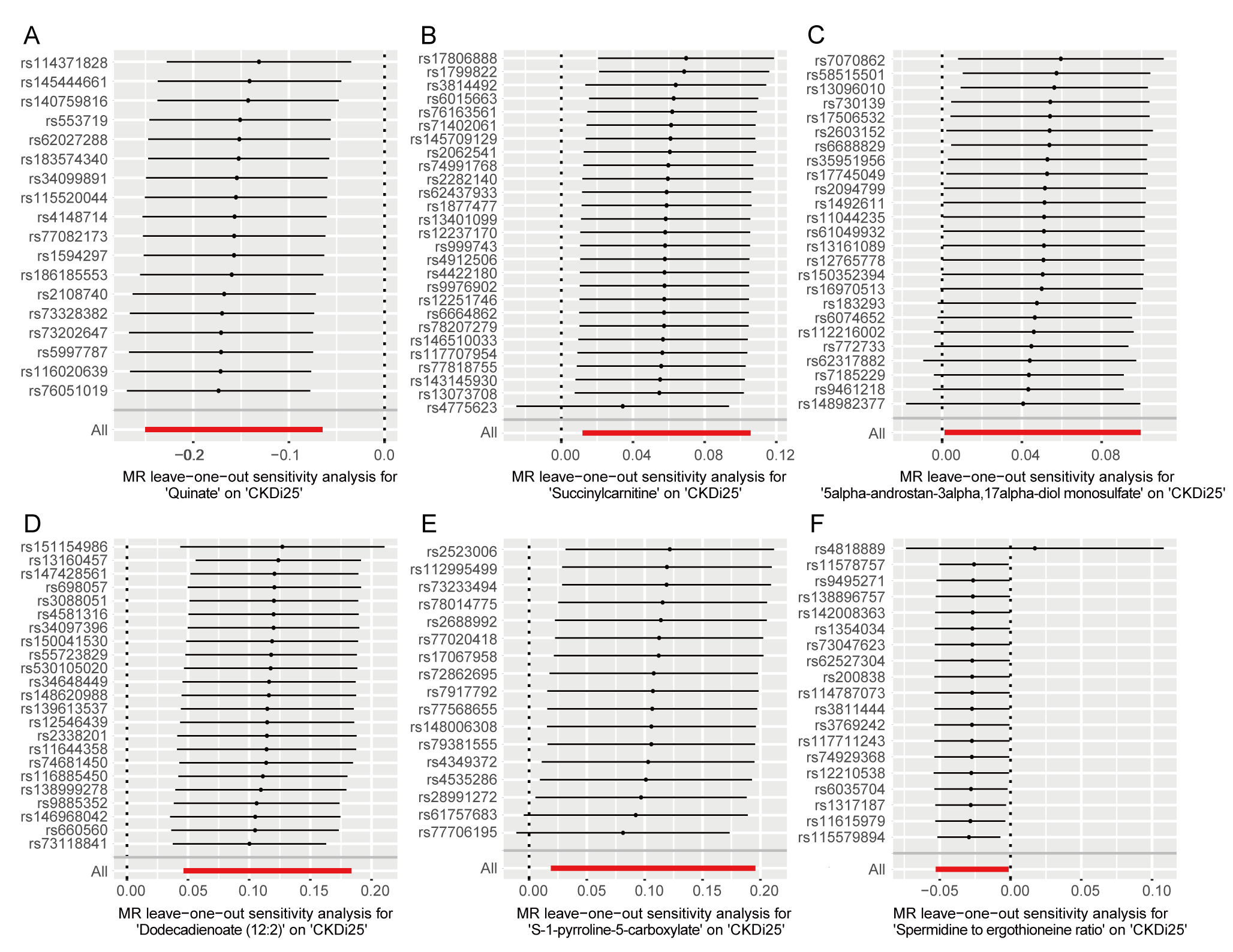


Supplement Figure S8. MR leave-one-out sensitivity analysis of serum metabolites -associated SNPs with risk of CKDi25.


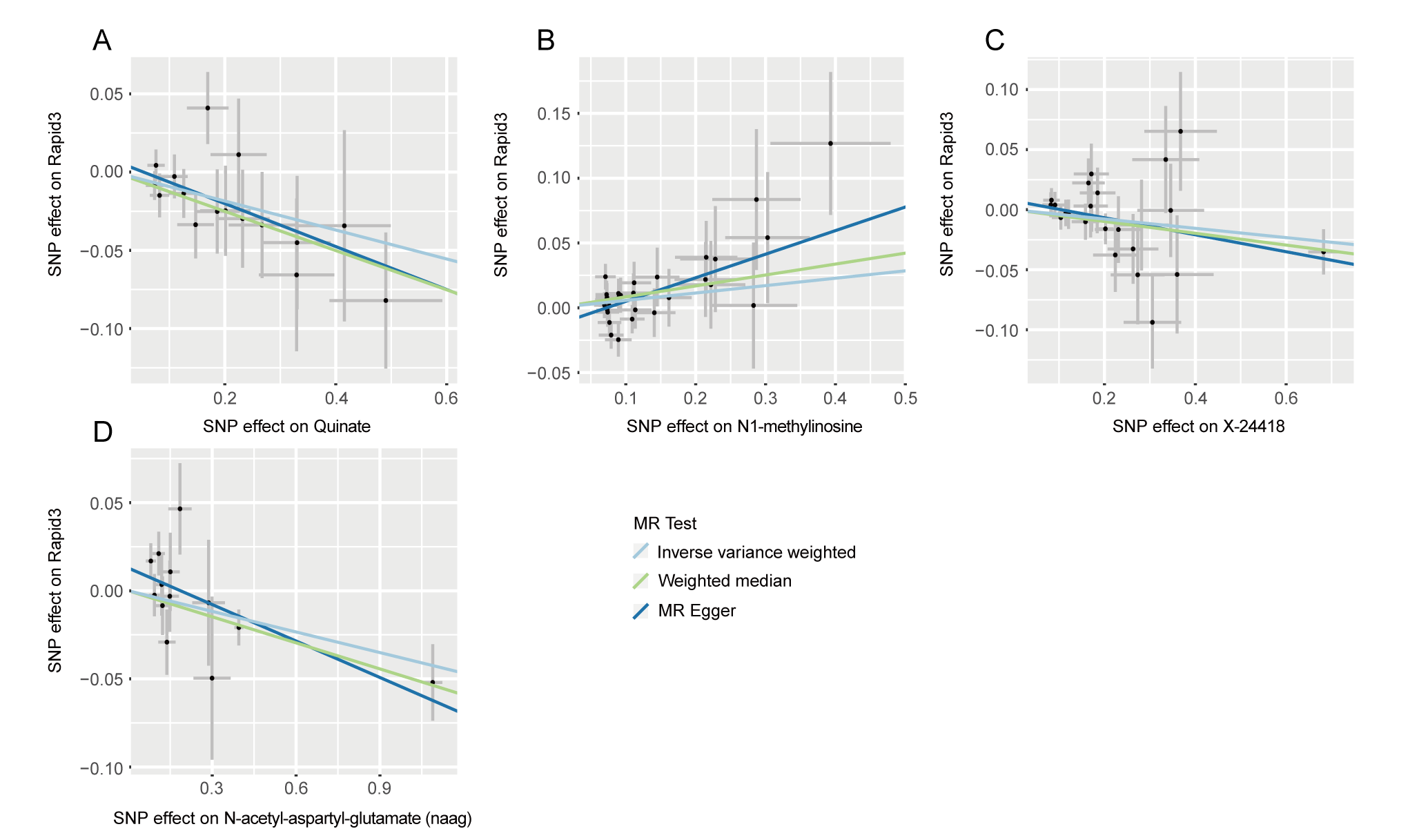


Supplement Figure S9. Scatter plot of the MR analysis results for the effect of the serum metabolites on Rapid3.


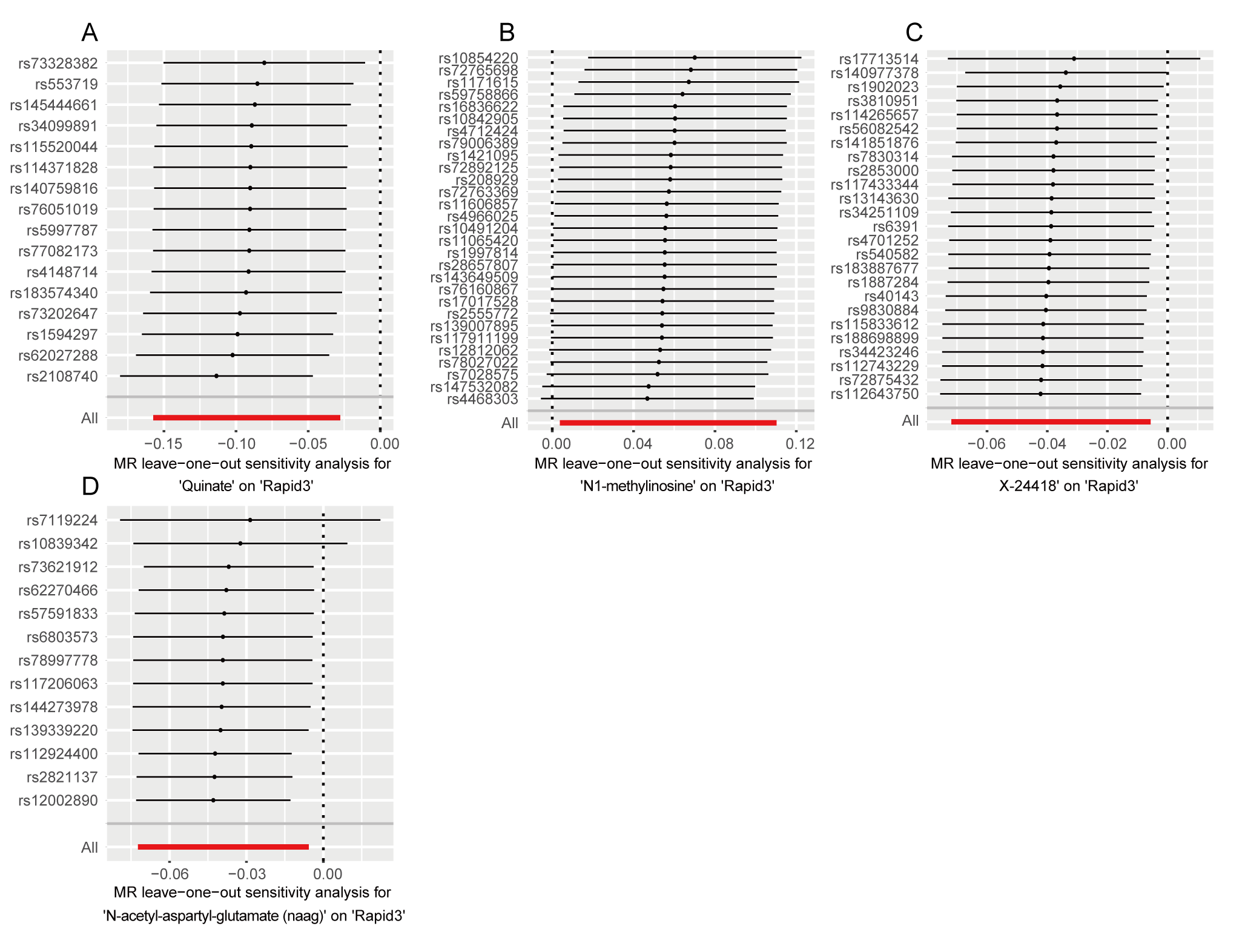


Supplement Figure S10. MR leave-one-out sensitivity analysis of serum metabolites -associated SNPs with risk of Rapid3.


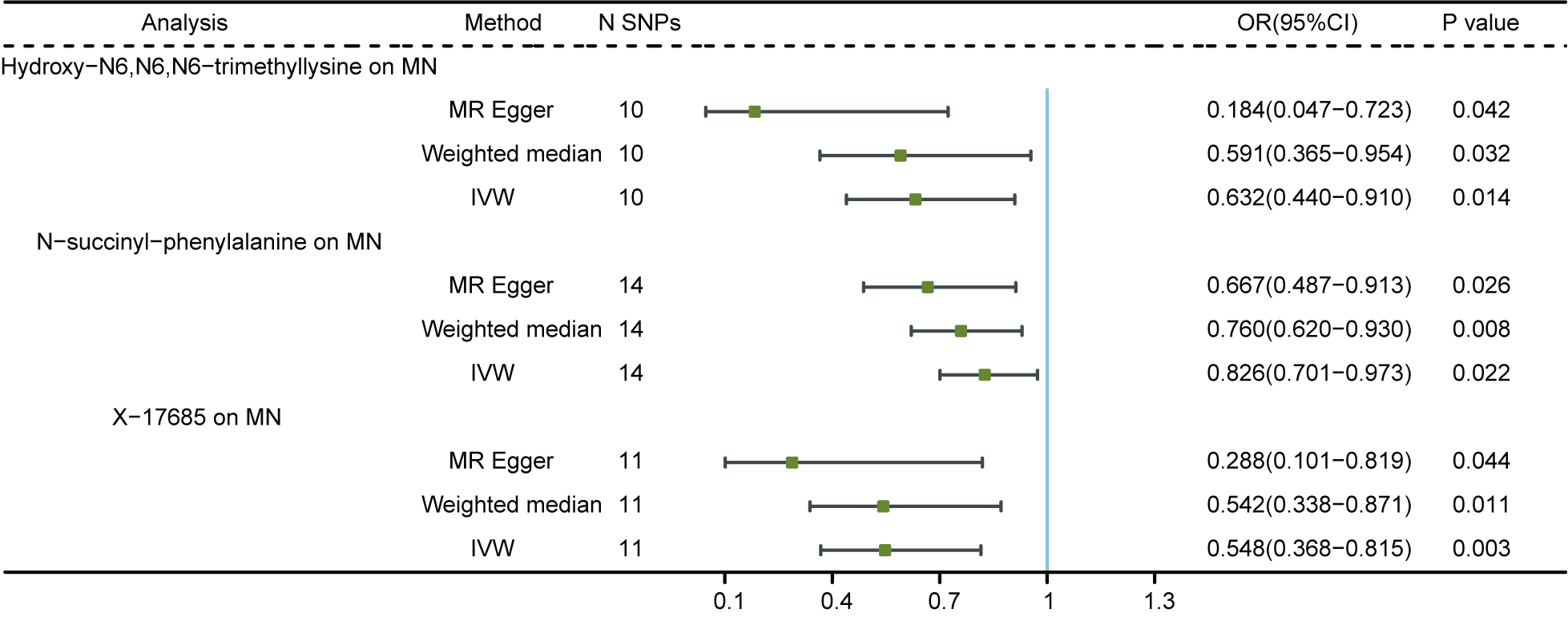


Supplement Figure S11. MR analysis of the causality of serum metabolites on MN. MN, membranous nephropathy; IVW, inverse variance weighted; SNP, single nucleotide polymorphism; OR, odds ratio; CI, confidence interval.


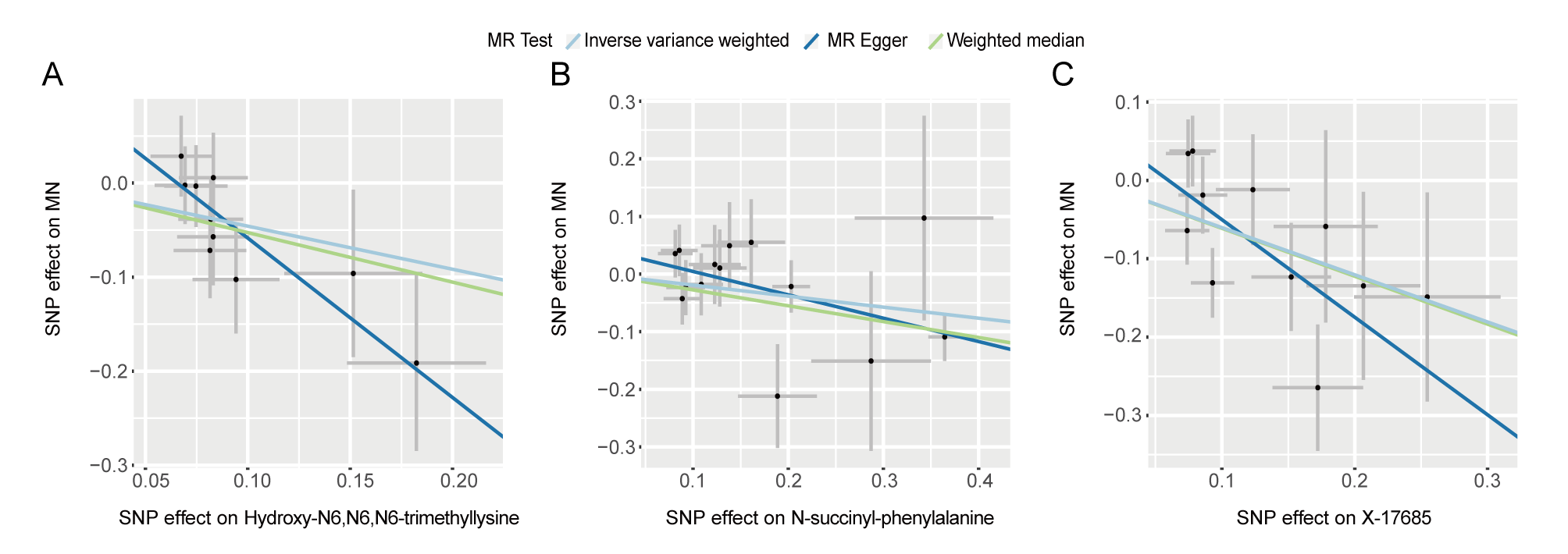


Supplement Figure S12. Scatter plot of the MR analysis results for the effect of the serum metabolites on **MN**.


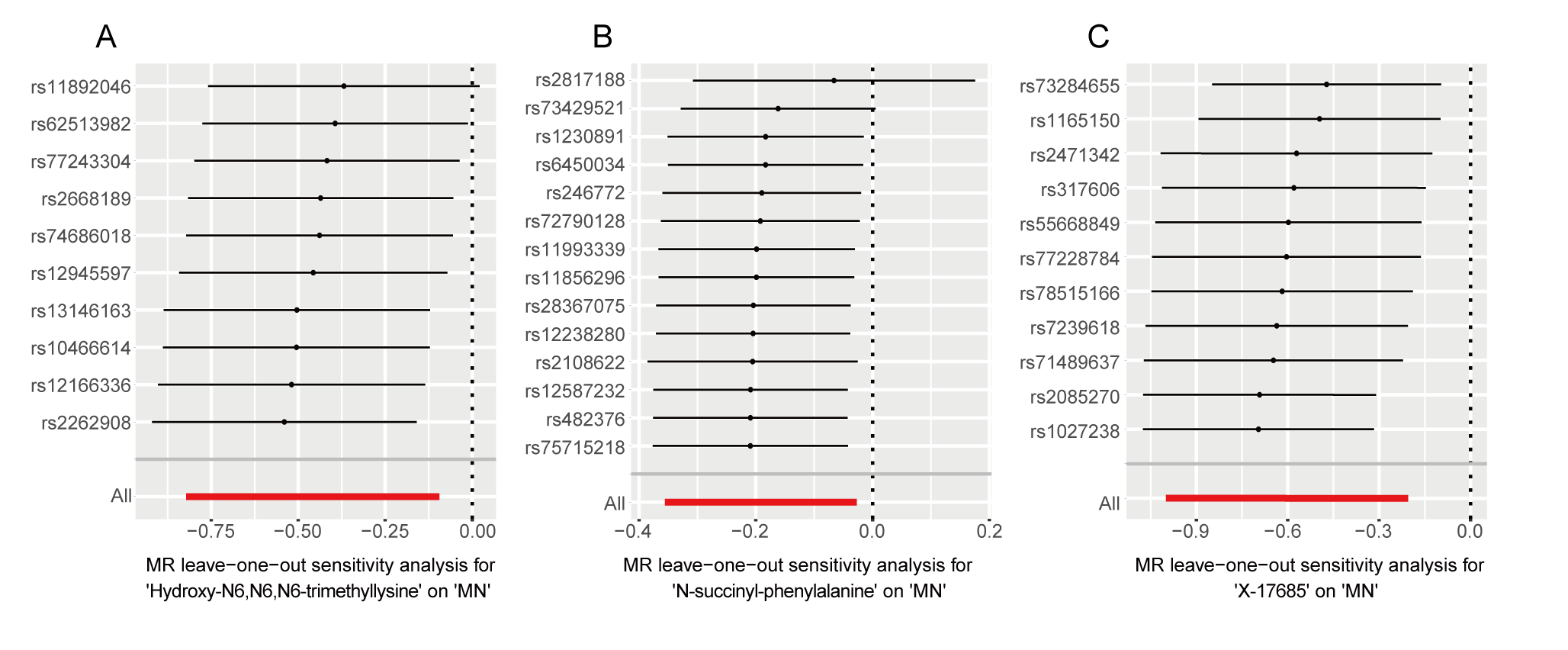


Supplement Figure S13. MR leave-one-out sensitivity analysis of serum metabolites -associated SNPs with risk of MN.


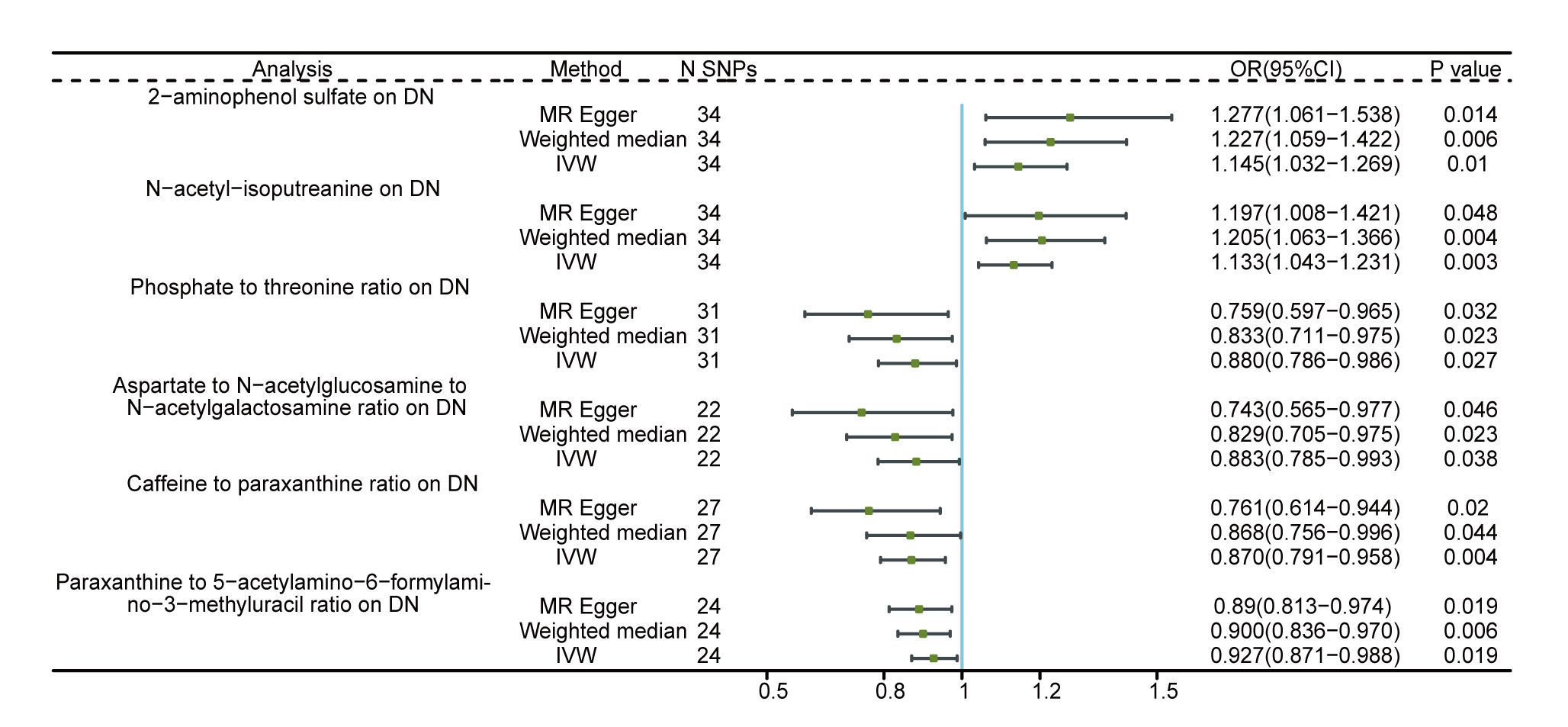

Supplement Figure S14. MR analysis of the causality of serum metabolites on DN. DN, diabetic nephropathy; IVW, inverse variance weighted; SNP, single nucleotide polymorphism; OR, odds ratio; CI, confidence interval.


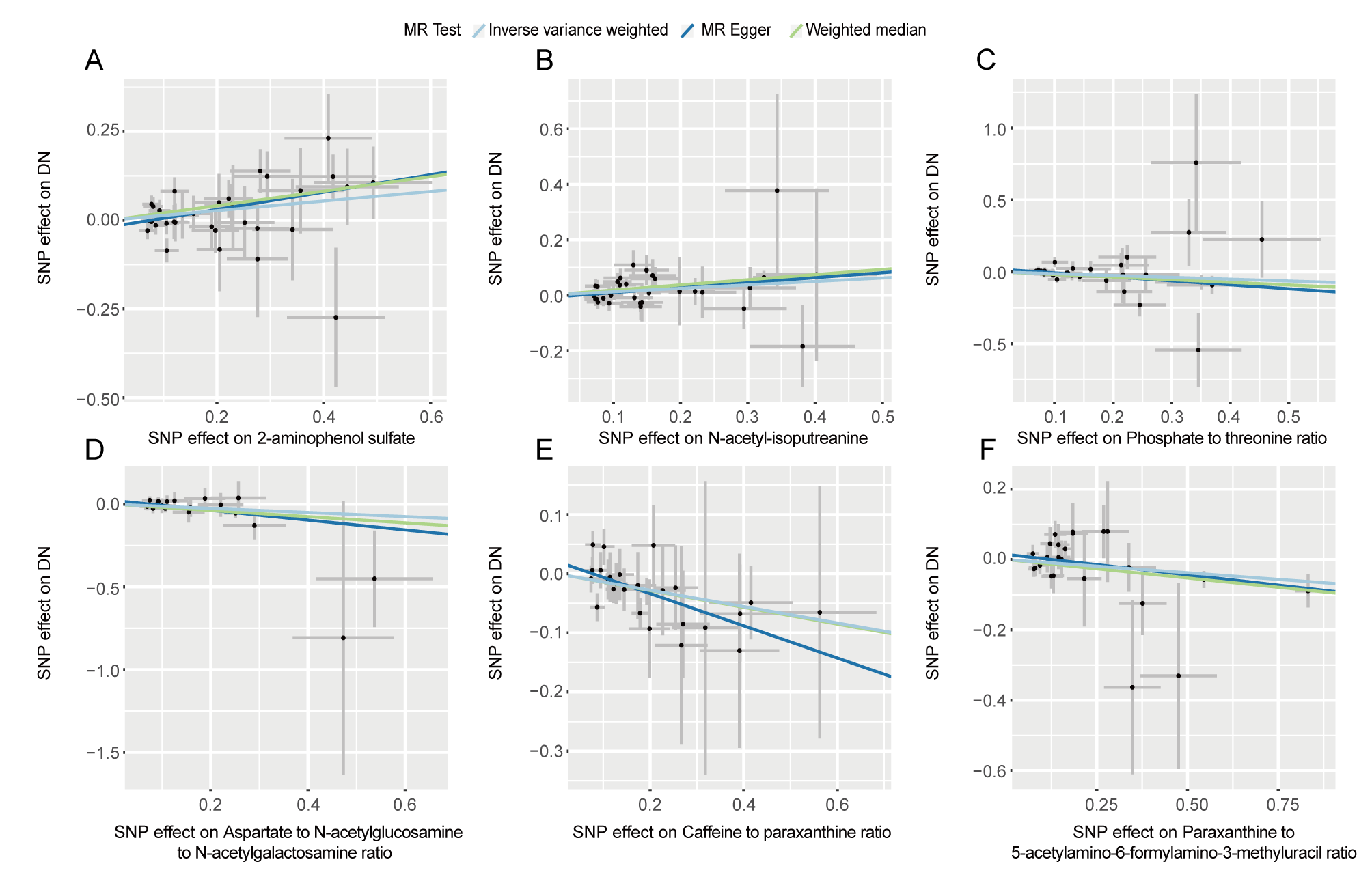


Supplement Figure S15. Scatter plot of the MR analysis results for the effect of the serum metabolites on DN.


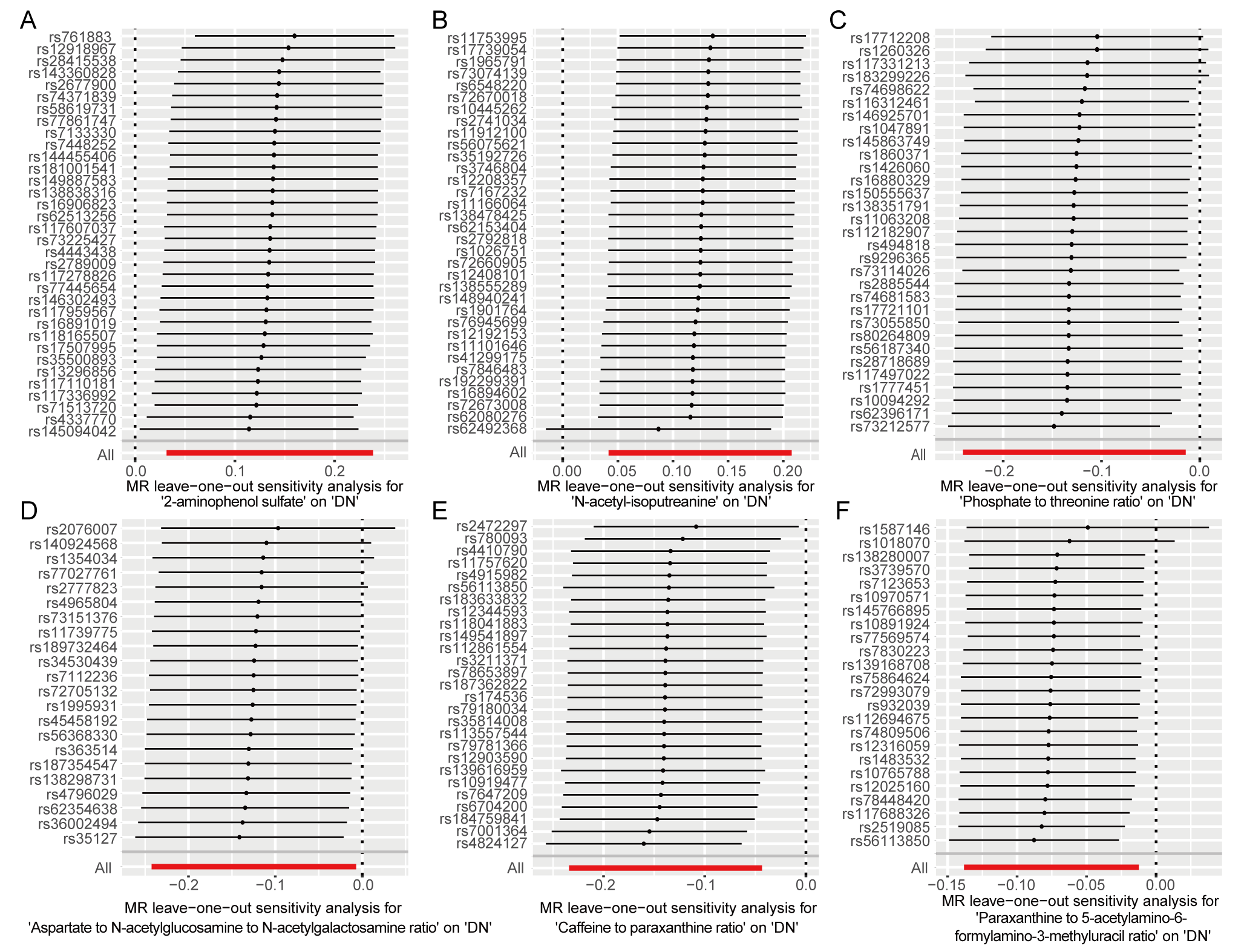


Supplement Figure S16. MR leave-one-out sensitivity analysis of serum metabolites -associated SNPs with risk of DN.


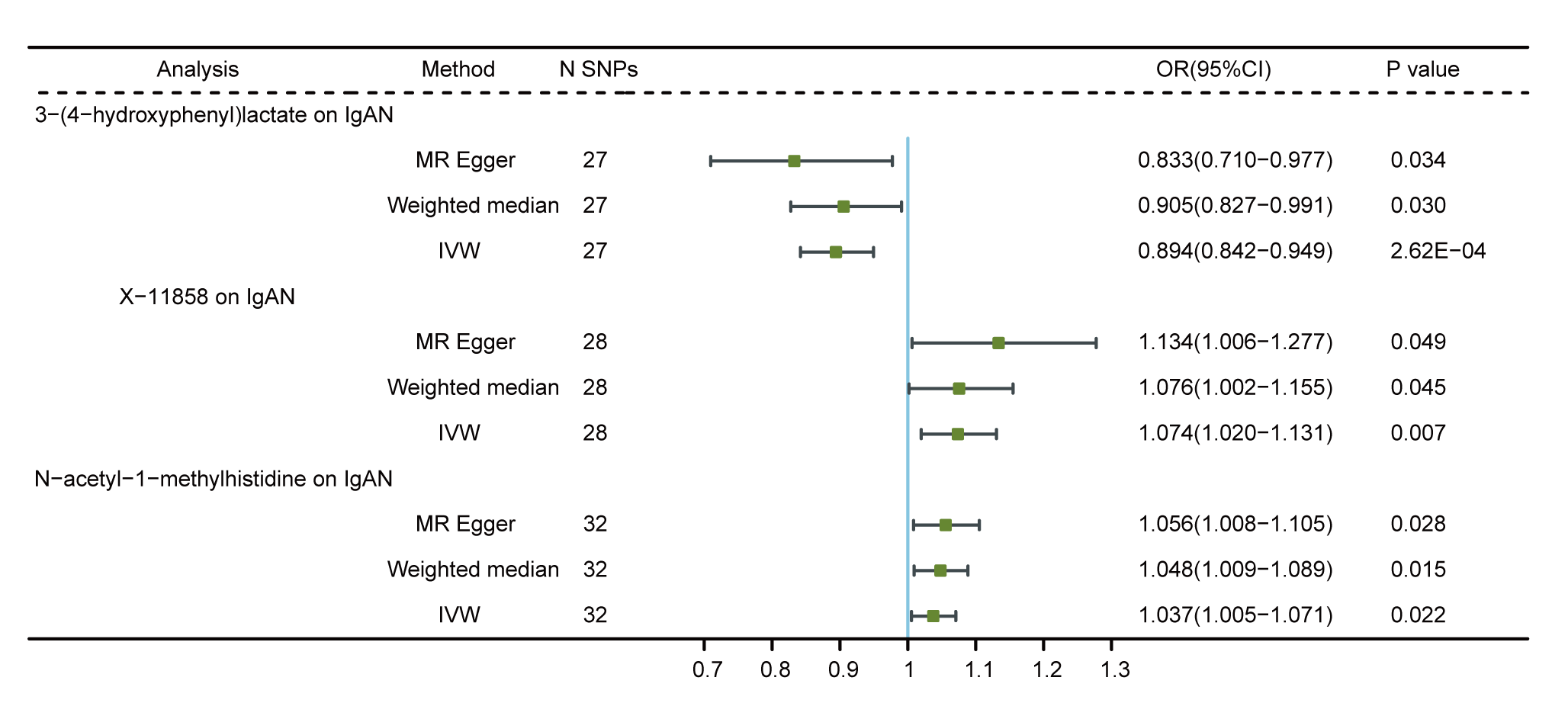


Supplement Figure S17. MR analysis of the causality of serum metabolites on IgAN. IgAN, immunoglobulin A nephropathy; IVW, inverse variance weighted; SNP, single nucleotide polymorphism; OR, odds ratio; CI, confidence interval.


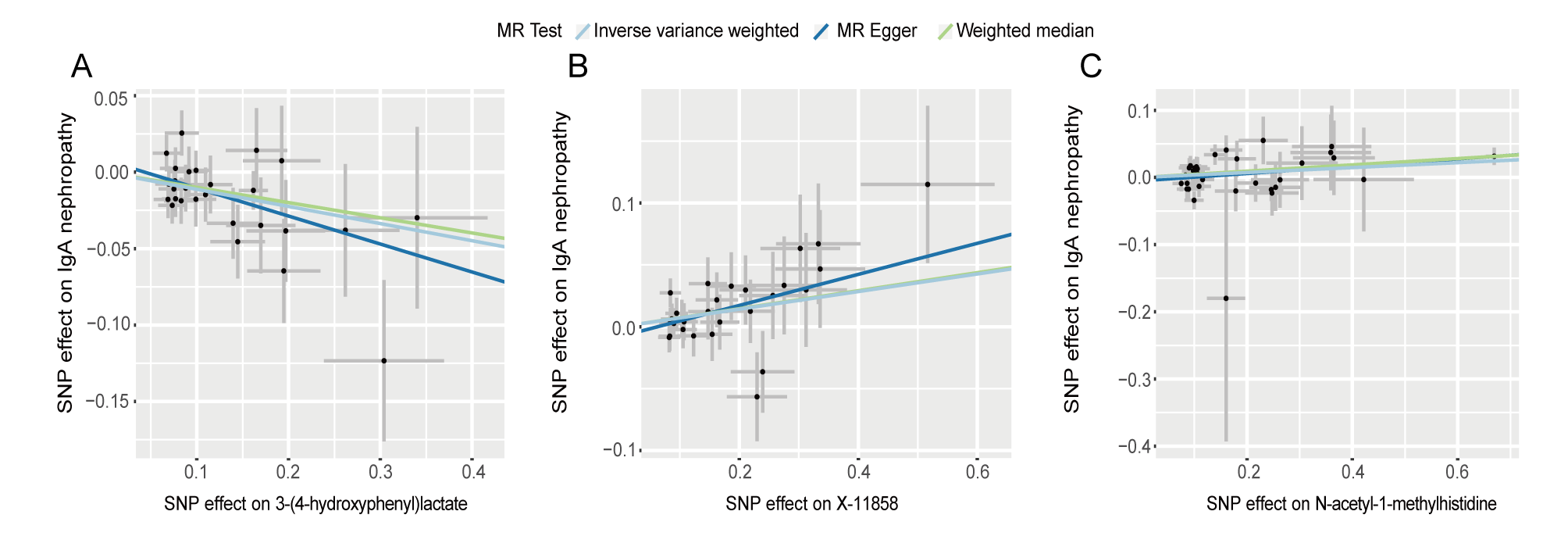


Supplement Figure S18. Scatter plot of the MR analysis results for the effect of the serum metabolites on IgAN.


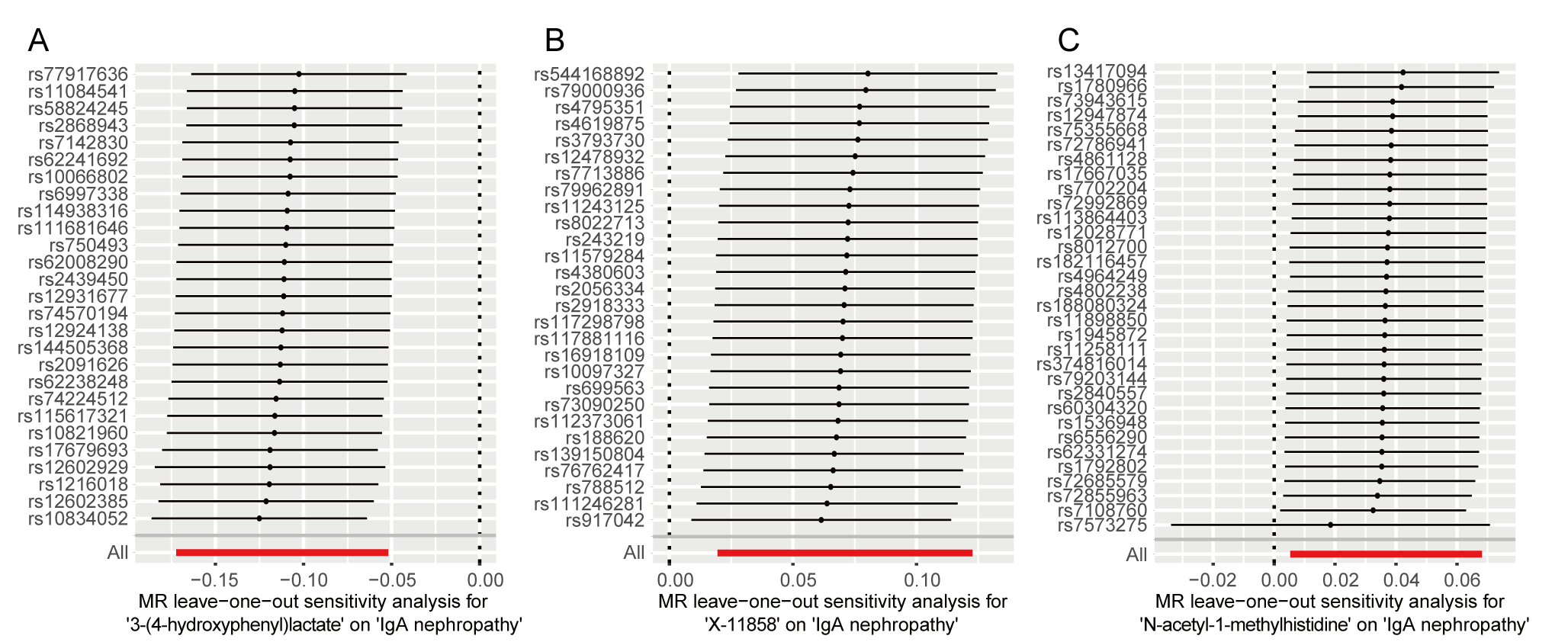


Supplement Figure S19. MR leave-one-out sensitivity analysis of serum metabolites -associated SNPs with risk of IgAN.
